# Caveolin-1 Promotes Early Neuronal Maturation via Caveolae-Independent Trafficking of N-Cadherin and L1

**DOI:** 10.1016/j.isci.2018.08.014

**Published:** 2018-08-21

**Authors:** Mima Shikanai, Yoshiaki V. Nishimura, Miwa Sakurai, Yo-ichi Nabeshima, Michisuke Yuzaki, Takeshi Kawauchi

**Affiliations:** 1 Department of Physiology, Keio University School of Medicine, 35 Shinanomachi, Shinjuku-ku, Tokyo 160-8582, Japan; 2 Division of Neuroscience, Faculty of Medicine, Tohoku Medical and Pharmaceutical University, 1-15-1 Fukumuro, Miyaginoku, Sendai, Miyagi 983-8536, Japan; 3 Laboratory of Molecular Life Science, Institute of Biomedical Research and Innovation, Foundation for Biomedical Research and Innovation at Kobe (FBRI), 2-2 Minatojima-Minamimachi Chuo-ku, Kobe 650-0047, Japan; 4 Precursory Research for Embryonic Science and Technology (PRESTO), Japan Science and Technology Agency (JST), Saitama 332-0012, Japan

**Keywords:** Molecular Neuroscience, Developmental Neuroscience, Cellular Neuroscience

## Abstract

Axon specification is morphologically reproducible *in vitro*, whereas dendrite formation differs *in vitro* and *in vivo*. Cortical neurons initially develop immature neurites, but *in vivo* these are eliminated concurrently with the formation of a leading process, the future dendrite. However, the molecular mechanisms underlying these neuronal maturation events remain unclear. Here we show that caveolin-1, a major component of caveolae that are never observed in neurons, regulates *in vivo*-specific steps of neuronal maturation. Caveolin-1 is predominantly expressed in immature cortical neurons and regulates clathrin-independent endocytosis. *In vivo* knockdown of caveolin-1 disturbs immature neurite pruning, leading process elongation, and subsequent neuronal migration. Importantly, N-cadherin and L1, which are required for immature neurite formation, undergo caveolin-1-mediated endocytosis to eliminate immature neurites. Collectively, our findings indicate that caveolin-1 regulates N-cadherin and L1 trafficking independent of caveolae, which contributes to spatiotemporally restricted cellular events; immature neurite pruning and leading process elongation during early neuronal maturation.

## Highlights

• Clathrin-independent endocytosis regulates early neuronal maturation
• Caveolin-1 promotes immature neurite pruning and leading process elongation *in vivo*
• Caveolin-1 controls N-cadherin and L1 trafficking independent of caveolae
• Suppression of caveolin-1 results in defects in cortical neuronal migration

## Graphical Abstract

**Figure:**
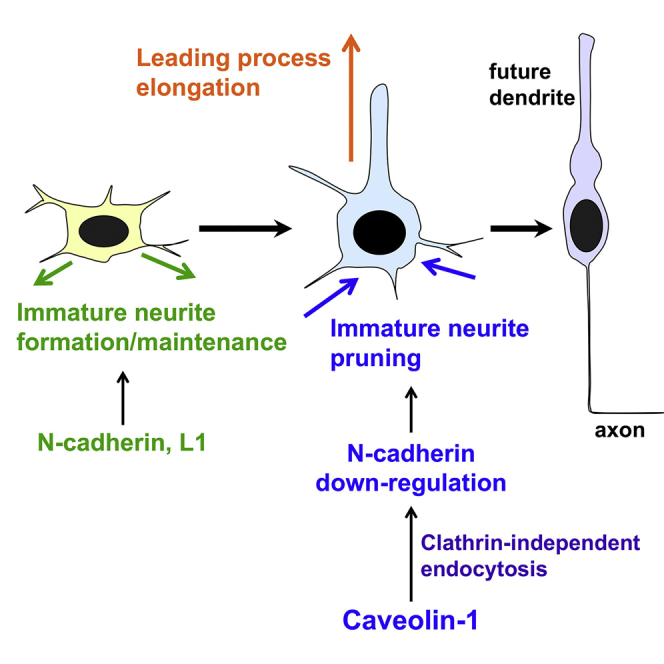

## Introduction

Neurons are highly polarized cells that have two functionally distinct cellular compartments: an axon, which generates and transduces action potentials, and dendrites, which receive and integrate electrical inputs from synapses. Establishment of the neural networks consisting of axons and dendrites elongated from the systematically allocated neuronal cell bodies is the basis of higher order brain functions. Axon specification is morphologically reproducible *in vitro,* and many molecules regulating axon formation have been identified (Arimura and Kaibuchi, 2007, Govek et al., 2018, Namba et al., 2014, Yi et al., 2010). In contrast, dendrite formation differs *in vitro* and *in vivo*.

Cultured cortical and hippocampal neurons extend many immature neurites, which directly mature into dendrites *in vitro* (Dotti et al., 1988). In the developing cerebral cortex, newly generated neurons also exhibit multipolar morphologies with multiple immature neurites. However, unlike *in vitro-*cultured neurons, cortical neurons eliminate the immature neurites after axon specification and concurrently form a leading process (Ehlers and Polleux, 2010, Kawauchi, 2015, Marin et al., 2010). The newly formed bipolar neurons, with a leading process and an axon, are called locomoting neurons and migrate long distances toward the pial surface. At the final phase of the neuronal migration, the leading process becomes branched and matures into dendrites (Hatanaka and Murakami, 2002). Thus, in the developing cerebral cortex, the dendrites originate from a leading process, rather than immature neurites. Although several molecules that control dendrite branching, an event that occurs at the late phase of neuronal maturation, have been identified (de Anda et al., 2012, Lefebvre et al., 2015), molecular and cellular mechanisms for *in vivo-*specific events, such as immature neurite pruning and leading process elongation, are poorly understood.

Cellular morphological changes rely on not only cytoskeletal organization but also endocytosis and membrane trafficking. Endocytosis can be classified into many types, including clathrin-mediated and caveolae-mediated endocytosis (Doherty and McMahon, 2009). A small GTPase, Rab5, controls endocytosis and trafficking to the early endosomes (Stenmark, 2009, Zerial and McBride, 2001). We previously reported that Rab5-dependent endocytic pathways play essential roles in the long-distance migration of the locomoting neurons in the developing cerebral cortex (Kawauchi et al., 2010). The functional suppression of clathrin heavy chains (HCs) leads to a neuronal migration defect similar to Rab5 knockdown, suggesting that clathrin-mediated endocytosis is required for neuronal migration (Shieh et al., 2011).

In contrast, classical caveolae-mediated endocytosis does not occur in neurons because neurons lack caveolae, flask- or omega-shaped membrane invaginations (Cheng and Nichols, 2016, Parton and del Pozo, 2013, Rakic, 1972, Shieh et al., 2011). However, *caveolin-1* (*Cav1*), which encodes a major component of caveolae, also has caveolae-independent functions (Echarri and Del Pozo, 2015, Gioiosa et al., 2008, Head and Insel, 2007, Takayasu et al., 2010, Trushina et al., 2006a) and is reported as a risk gene for schizophrenia (Allen et al., 2011, Kassan et al., 2017). A relationship between caveolin-1 and mutant Huntington disease protein (mHtt) has also been reported (Trushina et al., 2006b). Considering that schizophrenia is a neurodevelopmental disorder and that the expression of mHtt disturbs neuronal migration in the developing cerebral cortex (Barnat et al., 2017), it implies that caveolin-1 may have a role in neural development.

In this study, we found that unlike clathrin adaptor proteins, caveolin-1 is predominantly expressed in immature neurons in the developing cerebral cortex and promotes both immature neurite pruning and leading process elongation through the internalization of N-cadherin (*Cdh2*) and L1 (*L1cam*) cell adhesion molecules, suggesting that clathrin-independent endocytosis is required for spatiotemporally restricted cellular events *in vivo*. These data indicate that caveolin-1-mediated endocytosis regulates *in vivo*-specific steps of the early phase of neuronal maturation.

## Results

### Caveolin-1 Is Predominantly Expressed in Immature Neurons

**Figure 1:**
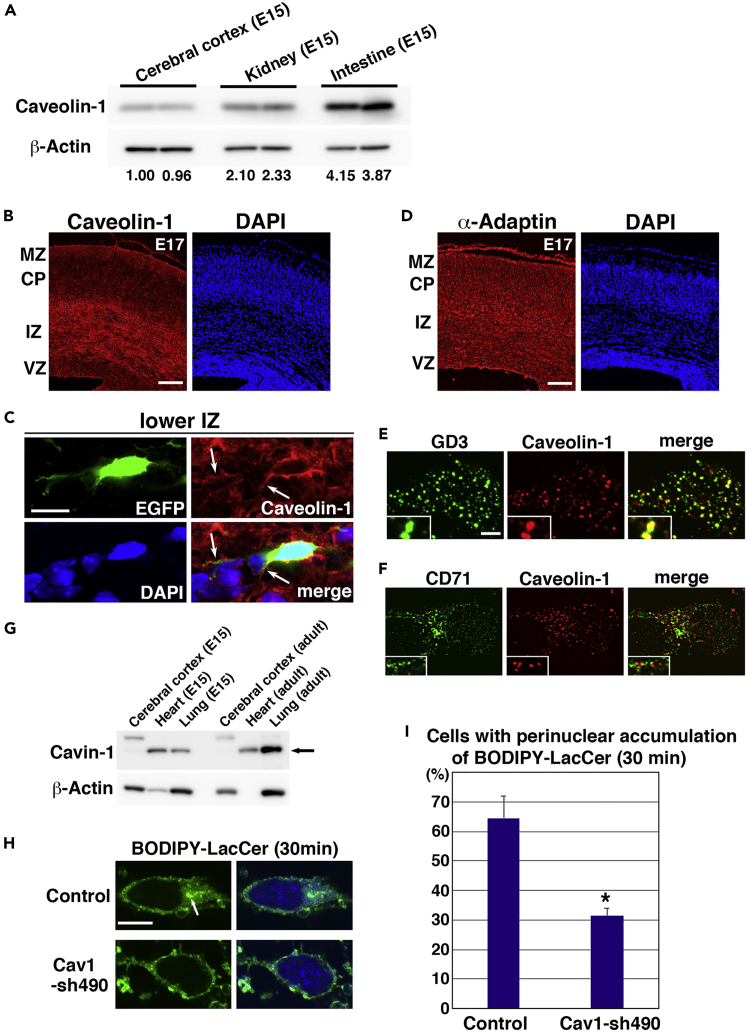
Caveolin-1 Is Strongly Expressed in the Immature Cortical Neurons and Required for Clathrin-Independent Endocytosis in Neurons (A) Immunoblot analyses of lysates from the indicated tissues at E15 with the indicated antibodies. The ratios of immunoblot band intensities of caveolin-1/β-actin, as determined by Las-3000mini (Fuji-film), are indicated. See also Figure S1. (B–D) Cryosections of cerebral cortices at E17 were immunostained with anti-caveolin-1 (B and C) or anti-α-adaptin (D) and DAPI (nuclear staining). (C) High magnification images near the lower intermediate zone. White arrows indicate immature neurites. MZ, marginal zone; CP, cortical plate; IZ, intermediate zone; VZ, ventricular zone. See also Figure S2. (E) Primary cortical neurons from E15 cerebral cortices incubated for 2 days *in vitro* and stained with anti-GD3 (green) and anti-caveolin-1 (red) antibodies. Anti-GD3 antibody may induce the clustering of GD3 after fixation (because lipids are not fixed by 4% PFA), and a part of caveolin-1 is also observed in the GD3-containing small aggregates on the membrane, suggesting that caveolin-1 may interact with GD3 and form the co-aggregates with GD3 after fixation. See also Figure S3. (F) Primary cortical neurons from E15 cerebral cortices incubated for 2 days *in vitro* and stained with anti-CD71 (green) and anti-caveolin-1 (red) antibodies. See also Figure S3. (G) Immunoblot analyses of lysates from the indicated tissues (E15 or adult mice) with anti-Cavin-1 (black arrow) and anti-β-actin antibodies. (H and I) (H) Primary cortical neurons from E15 cerebral cortices were transfected with CAG = ECFP-Mem (blue, a marker for transfected cells), incubated for 2 days *in vitro,* and treated with BODIPY-LacCer for 30 min before fixation. White arrows indicate the perinuclear accumulation of BODIPY-LacCer (green). The graph in (I) shows the ratio of cells with perinuclear accumulation of BODIPY-LacCer. *n* = 3 (control: 82 cells, Cav1-sh490: 87 cells). Each score represents the mean of ratios ± SEM. Significance compared with control was determined by Student's *t* test (p = 0.0142) and Mann-Whitney's *U* test (p = 0.0495). *p < 0.05. See also Figures S4 and S5. Scale bars: 100 μm in (B), 10 μm in (C), 100 μm in (D), 2 μm in (E and F), and 3 μm in (H).

Caveolin-1 expression has been reported to be low in the brain (Cameron et al., 1997, Head and Insel, 2007, Scherer et al., 1996). Consistent with this, low protein levels of caveolin-1 were detected in the embryonic cerebral cortex at embryonic day 15 (E15), which were about 40% and 20% of its levels in E15 kidney and intestine, respectively (Figure 1A), whereas other membrane-associated proteins, including clathrin HC and Rab5, were strongly expressed in E15 cerebral cortex (Figure S1A). A second antibody for caveolin-1 was also used to verify the expression of caveolin-1 in the embryonic cerebral cortex (Figures S1B and S1C). This suggests that only a subpopulation of neurons expresses caveolin-1.

To test this, we performed immunohistochemical analyses in mouse cerebral cortices at E17. Caveolin-1 proteins were mainly detected in the intermediate zone (IZ) and marginal zone of E17 cerebral cortices, but low levels were detected in the ventricular zone (VZ) and cortical plate (CP) (Figure 1B). The IZ is a cell-sparse area, mainly populated by immature neurons with multipolar and locomoting morphologies and axon bundles of mature and immature neurons. High-magnification images show that caveolin-1 is expressed in the immature neurites and cell soma of multipolar neurons and the soma of the locomoting neurons in the IZ (Figures 1C and S2A), whereas very low levels of caveolin-1 expression were observed in the axon bundles (Figure S2B). Caveolin-1 was barely detectable in the locomoting neurons in the CP (Figure S2A). This suggests that caveolin-1 expression is transiently increased in the immature neurons in the IZ, but not in the CP, although a subset of mature neurons appear to re-express caveolin-1. In contrast, α-adaptin, a component of the AP-2 clathrin adaptor complex, was diffusely expressed throughout the cerebral cortex at E17 (Figure 1D).

We next examined the subcellular localization of caveolin-1 in immature primary cortical neurons at 2 days *in vitro*. As previously reported (Ariotti and Parton, 2013), caveolin-1 was localized at both the plasma membrane and the early endosomes, as visualized with transfected plasma membrane-targeted monomeric Azami Green 1 (PM-mAG1) and an early endosome marker, APPL1, respectively (Figures S3A and S3B). Caveolin-1 was found to partially colocalize with the GD3 ganglioside-rich membrane domain (Figures 1E and S3G). In contrast, no colocalization was observed with a non-raft marker (CD71) or with a tetraspanin protein (CD151) (Figures 1F, S3C, and S3G). Little to no colocalization of caveolin-1 was observed with α-adaptin, clathrin HC, and Ror1, a receptor tyrosine kinase that is involved in clathrin-mediated endocytosis of Frizzled2 (Sato et al., 2010) (Figures S3D–S3G). Because Ror1 is also known to sustain caveolae structures through the interaction with Cavin-1 and caveolin-1 in non-neuronal cells (Yamaguchi et al., 2016), the different localization of Ror1 and caveolin-1 in immature neurons may support the notion that neurons lack caveolae.

### Involvement of Caveolin-1 in Endocytosis in Cortical Neurons

Previous electron microscopic analyses revealed that caveolae are not observed in migrating immature neurons in the embryonic cerebral cortex (Rakic, 1972, Shieh et al., 2011). Consistent with this, expression levels of cavin-1, a molecule required for the formation of caveolae, were very low in the embryonic cerebral cortex at E15 (Figure 1G). To examine caveolae-independent roles of caveolin-1 in immature neurons, we constructed a short hairpin RNA (shRNA) expression vector targeting the coding sequence of mouse *caveolin-1* (Cav1-sh490). When expressed in primary cortical neurons, Cav1-sh490 efficiently suppressed endogenous caveolin-1 expression (Figure S4A). In immature neurons *in vivo*, the reduced levels of caveolin-1 expression were hard to detect in low-magnification images of the Cav1-sh490-electroporated cerebral cortex, because caveolin-1 localizes near the membrane, but not the nucleus, and therefore it is hard to distinguish the staining of caveolin-1 that is derived from the electroporated and non-electroporated cells (Figures S5A and S5B). In contrast, this can be observed more clearly at higher magnification of the single immature neurons (Figures S4B and S5C).

We next examined the effect of caveolin-1 knockdown on endocytosis by using BODIPY-LacCer, a potential marker for clathrin-independent and caveolin-1-mediated endocytosis (Singh et al., 2003, Trushina et al., 2006b). At 30 min after treatment, most of the BODIPY-LacCer was internalized and transported to the perinuclear regions in control neurons. In contrast, Cav1-sh490-expressing neurons exhibited defects in the uptake of BODIPY-LacCer (Figures 1H and 1I). A similar result was obtained when primary cortical neurons were treated with Alexa 555-conjugated cholera toxin B subunit (Pelkmans and Zerial, 2005, Shvets et al., 2015, Singh et al., 2003) (Figure S4C). Together with the observation that caveolin-1 is not colocalized with clathrin HC and α-adaptin, these data indicate that caveolin-1 is involved in clathrin-independent endocytosis in cortical neurons.

### Caveolin-1 Regulates *In Vivo* Neuronal Maturation

Using an *in vivo* gene transfer technique, *in utero* electroporation (Kawauchi et al., 2003), Cav1-sh490 was introduced into mouse embryonic cerebral cortices at E14. The electroporated cells were visualized with co-transfected EGFP. Except for short hairpin sequences, all genes were driven with a CAG promoter, unless otherwise specified.

At 46 hr after electroporation, control and Cav1-sh490-electroporated cells were mainly located at the VZ or subventricular zone, where neural progenitors exist. Both control and Cav1-sh490-electroporated cells normally expressed proliferative markers, phospho-histone H3 (PH3), and Ki67, suggesting that knockdown of caveolin-1 does not disturb the proliferation of neural progenitors (Figures S6A and S6B). Normal expression of Tuj1, a neuronal marker, and morphology of Nestin-positive fibers suggested that neuronal differentiation and the extension of radial fibers in Cav1-sh490-electroporated cells are not affected (Figures S4B and S6C).

**Figure 2:**
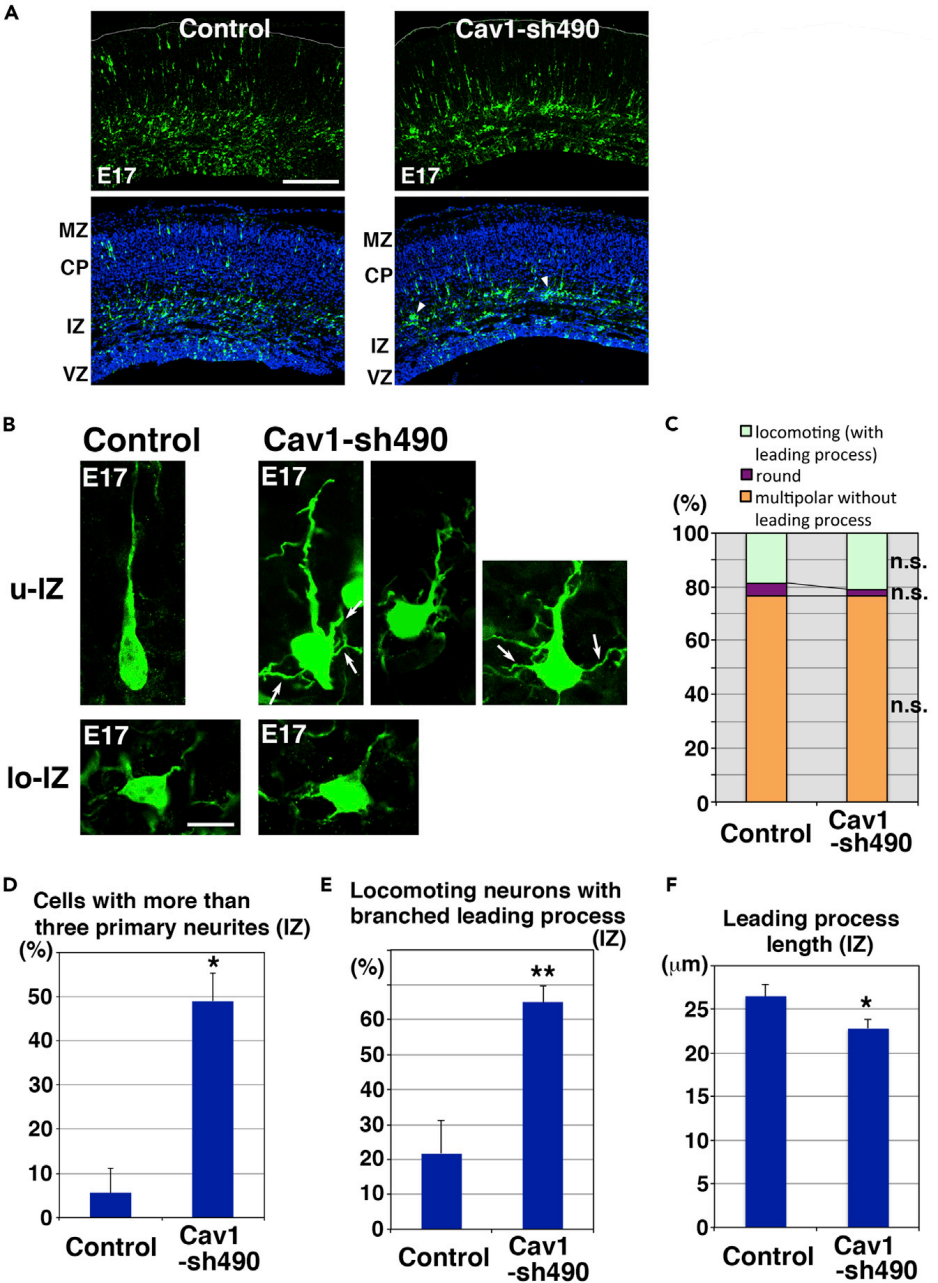
Caveolin-1 Is Required for Immature Neurite Pruning and Leading Process Elongation (A–F) Cerebral cortices at E17, electroporated with the indicated plasmids plus pCAG-EGFP at E14. (A) The lower panels show EGFP fluorescence (green) and nuclear staining with DAPI (blue). Arrowheads indicate small cell aggregates. (B) High-magnification images of the upper IZ (u-IZ) or lower IZ (lo-IZ) of the cerebral cortices. White arrows indicate abnormal primary neurites. (C) The ratio of cells with the indicated morphology in the IZ. Control and Cav1-sh490: *n* = 4 brains. No significant differences (n.s.) between control and Cav1-sh490-electroporated neurons were found by Mann-Whitney's *U* test and Student's *t* test (locomoting: p = 1 or 0.6148, round: p = 0.1489 or 0.1916, multipolar: p = 1 or 0.9484, respectively). (D and E) The ratio of locomoting neurons with more than three primary neurites (D) or branched leading processes (E) in the IZ. Control: *n* = 4 (52 cells) (D and E), Cav1-sh490: *n* = 5 (128 cells) (D) or 8 (171 cells) (E). Each score represents the mean of ratios ± SEM. Significance was determined by Mann-Whitney's *U* test [(D) p = 0.01431, (E) p = 0.006578) and Student's *t* test (D) p = 0.001542, (E) p = 0.0006317]. **p < 0.01, *p < 0.05. (F) Average leading process length of the locomoting neurons in the IZ. Control: *n* = 82 cells, Cav1-sh490: *n* = 85 cells. Each score represents the mean length ± SEM. Significance was determined by Welch's *t* test (p = 0.02834). *p < 0.05. See also Figures S6 and S7. Scale bars: 200 μm in (A) and 10 μm in (B).

At E17, the majority of control and Cav1-sh490-electroporated cells were located at the IZ, where caveolin-1 was strongly expressed (Figure 2A). At this stage, the neuronal positioning did not significantly differ between control and Cav1-sh490-electroporated neurons, although Cav1-sh490-electroporated cells were not diffusely located in the IZ, implying that cell-to-cell interactions between the multipolar-shaped immature neurons might be increased (arrowheads in Figure 2A).

In the lower part of the IZ (lo-IZ), Cav1-sh490-electroporated cells extended many immature neurites, similar to control multipolar neurons (Figures 2B and 2C). However, caveolin-1-knockdown locomoting neurons in the upper part of the IZ still extended many neurites even after forming a leading process, whereas control neurons retracted their immature neurites and formed a leading process resulting in bipolar or unipolar morphologies (Figure 2B). The superfluous neurites in the Cav1-sh490-electroporated neurons resulted from both abnormal branching of the leading processes and extension of excess primary neurites from the cell bodies (Figures 2D and 2E). Time-lapse imaging showed that control locomoting neurons retracted their immature neurites shortly after the leading process formation (Figure S7A). However, the Cav1-sh490-electroporated neurons retained their immature neurites for more than 6 hr after the leading process formation and were observed to retract and extend these neurites, a feature of immature neurites (Figure S7B).

The ratio of cells in the IZ with a leading process (locomoting) or without a leading process (round or multipolar) was not significantly different between control and Cav1-sh490-electroporated cortices, suggesting that initiation of the leading process formation occurs in the caveolin-1-knockdown neurons (Figure 2C). However, the leading process length of the Cav1-sh490-electroporated neurons in the IZ was significantly shorter than that of control (Figure 2F). These data indicate that caveolin-1 is required for the pruning of neurites in immature neurons and the stabilization of the bipolar morphology.

**Figure 3:**
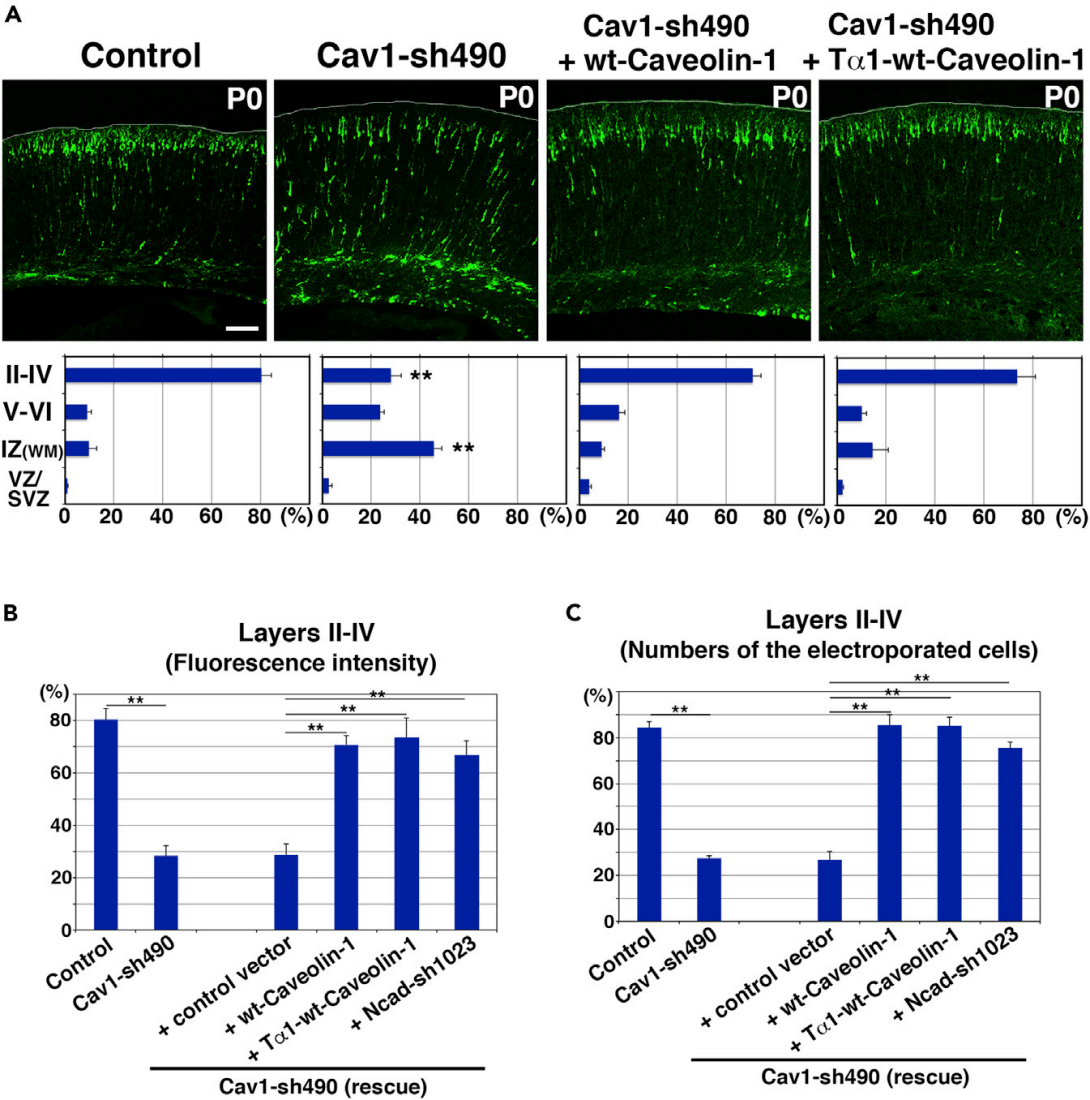
Knockdown of Caveolin-1 Results in Neuronal Migration Defects (A–C) Cerebral cortices at P0, electroporated with the indicated plasmids plus pCAG-EGFP at E14. The lower graphs in (A) show the estimation of cell migration, which was carried out by recording fluorescence intensities of EGFP in distinct regions of the cerebral cortices using Leica SP5 software. (B and C) The graphs show the ratio of the fluorescence intensities of EGFP (B) or the number of the electroporated cells (C) in the upper part of the cortical plate (future layers II–IV) to the whole cerebral cortices. Each bar represents the mean percentage of relative intensities ± SEM. Control: *n* = 6 brains, Cav1-sh490: *n* = 7 brains, Cav1-sh490 + CAG-wt-Caveolin-1: *n* = 8 brains, Cav1-sh490 + Tα1-wt-Caveolin-1: *n* = 6 brains. Significance compared with control was determined by Student's *t* test (A) or one-way ANOVA with post hoc Tukey-Kramer test (B and C). (A) Cav1-sh490 (layer II–IV) p = 0.000002286, Cav1-sh490 (IZ) p = 0.000007796. **p < 0.01. (B and C) ** p < the critical value at 1% (control versus Cav1-sh490, control versus Cav1-sh490 + control vector, Cav1-sh490 versus Cav1-sh490 + CAG-wt-caveolin-1, Cav1-sh490 versus Cav1-sh490 + Tα1-wt-caveolin-1, Cav1-sh490 versus Cav1-sh490 + Ncad-sh1023, Cav1-sh490 + control vector versus Cav1-sh490 + CAG-wt-caveolin-1, Cav1-sh490 + control vector versus Cav1-sh490 + Tα1-wt-caveolin-1, Cav1-sh490 + control vector versus Cav1-sh490 + Ncad-sh1023). II–IV, layers II–IV of the cortical plate; V–VI, layers V–VI of the cortical plate; IZ, intermediate zone; WM, white matter; SVZ/VZ, subventricular zone/ventricular zone. Scale bar: 100 μm in (A).

At postnatal day 0 (P0), 5 days after electroporation, most of the control EGFP-positive cells reached the superficial layer of the CP, suggesting that at this stage neuronal migration was almost completed, consistent with previous reports (Kawauchi et al., 2003, Kawauchi et al., 2006) (Figure 3A). However, substantial numbers of the Cav1-sh490-electroporated cells remained stalled at the IZ (Figure 3A). EGFP-positive cell numbers and fluorescence intensities in the upper layers of the CP (future layers II–IV) were significantly reduced in the Cav1-sh490-electroporated cortices, compared with control (Figures 3B and 3C). Importantly, co-expression of wild-type (wt) caveolin-1 restored the migration defects observed in the Cav1-490-electroporated cortices, indicating that these phenotypes are not due to off-targeting effects (Figures 3A–3C). In addition, co-expression of a neuron-specific Tα1 (α1-tubulin) promoter-driven wt caveolin-1 expression vector (Tα1-wt-caveolin-1) also rescued the migration defects of the Cav1-sh490-electroporated cells (Figures 3A–3C). These data indicate that caveolin-1 in neurons is required for proper positioning of the cortical neurons. Considering the weak expression of caveolin-1 in the locomoting neurons in the CP, the neuronal positioning defect may result from incomplete maturation and/or abnormally increased cell-to-cell adhesion of immature neurons in the IZ.

### N-Cadherin and L1 Are Cargo Molecules of Caveolin-1-Mediated Endocytic Pathways

**Figure 4:**
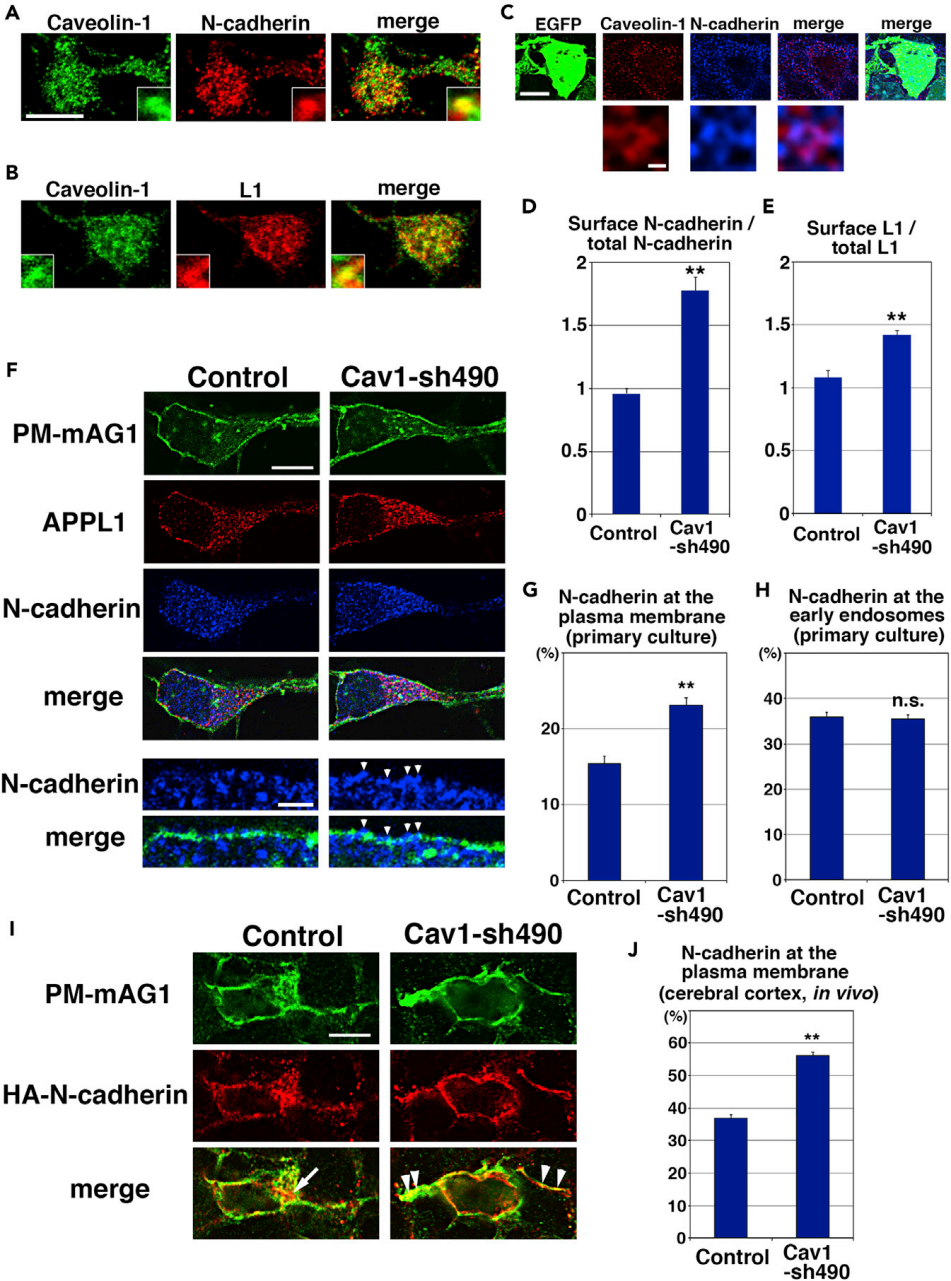
Caveolin-1 Promotes the Internalization of N-cadherin (A and B) Primary cortical neurons from E15 cerebral cortices incubated for 2 days *in vitro* and stained with anti-caveolin-1 (green) and anti-N-cadherin (red in A) or anti-L1 (red in B) antibodies. The images were obtained with TCS-SP5 (Leica). Insets are high magnification images. See also Figure S8. (C) Immature neurons in the intermediate zone of the cerebral cortices at E17, electroporated with pCAG-EGFP at E14. Frozen sections were immunostained with the indicated antibodies. The images were obtained with high-resolution microscopy (Nikon). The lower panels are high-magnification images. (D and E) The ratio of cell surface to total N-cadherin (D) or L1 (E) in primary cortical neurons (2 days *in vitro*). Control: *n* = 70 cells (D) or 53 cells (E), Cav1-sh490: *n* = 70 cells (D) or 56 cells (E). Each bar represents the mean ratio ± SEM. Significance compared with control was determined by Welch's *t* test [(D) p = 0.000000001292, (E) p = 0.000001138] and Mann-Whitney's *U* test [(D) p = 0.00000000006551, (E) p = 0.00000001977]. **p < 0.01. (F–H) (F) Primary cortical neurons from E15 cerebral cortices incubated for 2 days *in vitro* and stained with the indicated antibodies. Arrowheads indicate the accumulation of N-cadherin at the plasma membrane. The graphs show the ratio of the N-cadherin staining signals in the plasma membrane (G) and the APPL1-positive early endosomes (H) to total fluorescence intensities of N-cadherin. Control and Cav1-sh490: *n* = 18 cells (G) or 28 cells (H). Each bar represents the mean ratio ± SEM. Significance compared with control was determined by Student's *t* test [(G) p = 0.00006108, (H) P = 0.7428]. **p < 0.01; n.s., no significant differences. (I and J) (I) Immature neurons in the IZ of the cerebral cortices at E17, electroporated with the indicated plasmids plus pCAG-EGFP and pCAG-HA-N-cadherin at E14. Frozen sections were immunostained with the indicated antibodies. Arrow and arrowheads indicate the accumulation of HA-N-cadherin at the perinuclear regions and the plasma membrane in the immature neurites, respectively. (J) The graph shows the ratio of the HA-N-cadherin staining signals in the plasma membrane to total fluorescence intensities of HA-N-cadherin. Control and Cav1-sh490: *n* = 55 cells (J). Each bar represents the mean ratio ± SEM. Significance compared with control was determined by Student's *t* test (p = 7.021 × 10^−27^). **p < 0.01. Scale bars: 10 μm in (A and B), 4 μm in (upper panels in C), 0.2 μm in (lower panels in C), 4 μm in (upper panels in F), 1 μm in (lower panels in F), and 4 μm in (I).

We next searched for functional cargo molecules of caveolin-1-mediated endocytic pathways in neuronal maturation. We conducted a screen for transmembrane proteins that colocalize with caveolin-1 in cortical neurons and observed colocalization with N-cadherin and L1, classic cadherin family and immunoglobulin superfamily cell adhesion/recognition molecules, respectively, that are strongly expressed in embryonic neurons (Figures 4A, 4B, S1A, and S8B), but not with Ror1, CD151, and CD71 (Figure S3G). A small amount of N-cadherin was localized at the GD3-rich membrane domain (Figures S8A and S8B). In the immature neurons *in vivo*, endogenous caveolin-1 and N-cadherin were observed in the same vesicular compartments, although they did not completely overlap with each other (Figure 4C).

To examine whether caveolin-1 controls the trafficking of these candidates, Cav1-sh490-transfected cortical neurons were subjected to sequential immunocytochemical analyses. Before permeabilization, the fixed neurons were treated with anti-N-cadherin (extracellular region) antibody to visualize cell surface N-cadherin. Subsequently, the neurons were permeabilized with Triton X-100 and treated with another anti-N-cadherin antibody to stain with total N-cadherin. Ratios of the cell surface to total N-cadherin fluorescence intensities were measured and revealed that the ratio of cell surface N-cadherin was significantly increased in the Cav1-sh490-transfected neurons, compared with control (Figure 4D). Similarly, cell surface levels of L1 were also measured in primary cortical neurons. The ratio of surface to total L1 was found to be slightly increased (Figure 4E).

To confirm this, we quantified the N-cadherin staining signals in the plasma membrane (PM-mAG1-positive regions) and early endosomes (APPL1-positive regions). N-cadherin localization was increased at the PM-mAG1-positive regions in Cav1-sh490-transfected primary cortical neurons (Figures 4F and 4G) and immature neurons *in vivo* (Figures 4I and 4J), whereas it was unchanged at the APPL1-positive regions in the cortical neurons (Figure 4H). These data indicate that caveolin-1 is required for the internalization of N-cadherin and L1.

### N-Cadherin and L1 Are Required for Immature Neurite Formation

**Figure 5:**
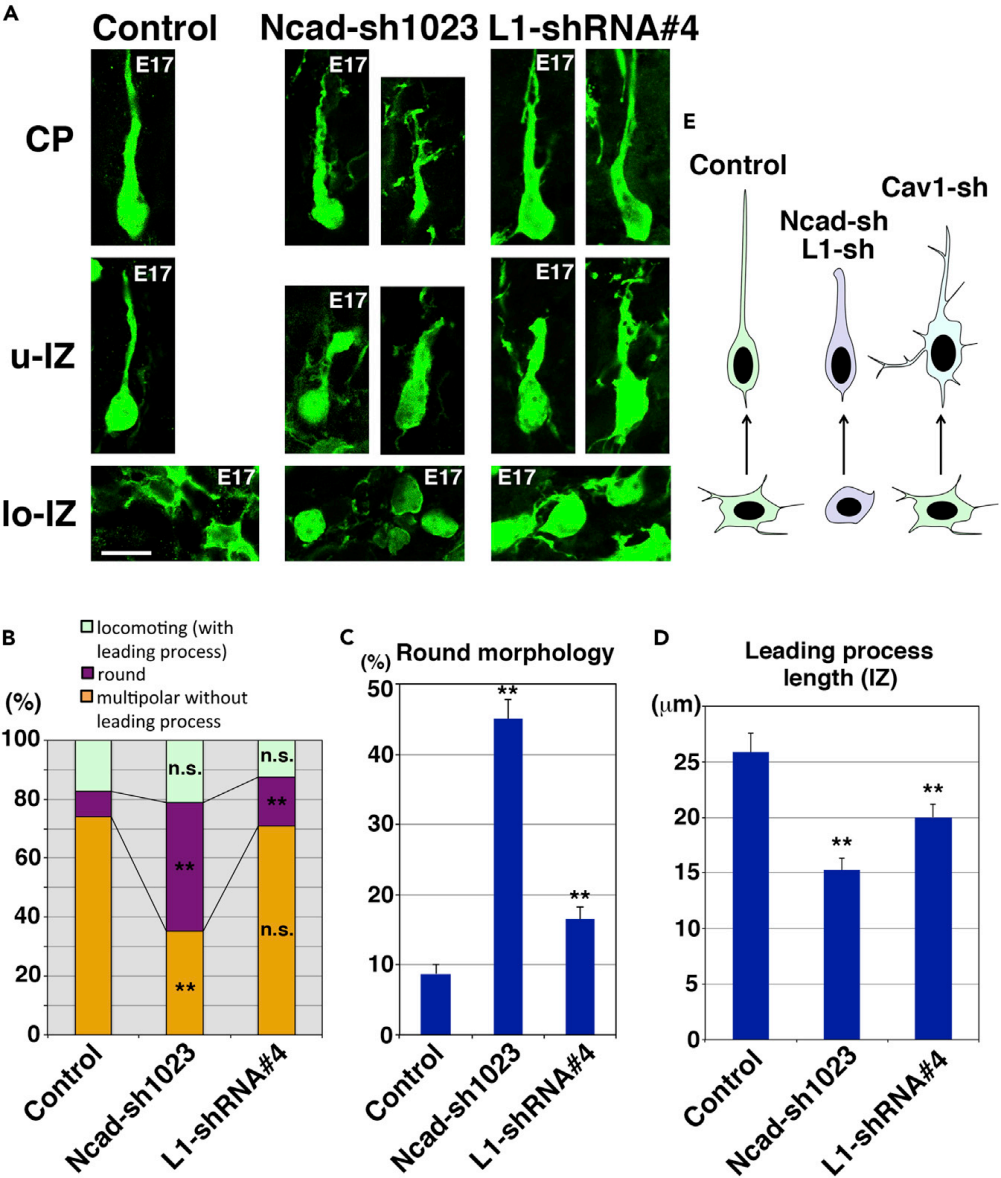
N-cadherin and L1 Are Required for the Immature Neurite Formation/Maintenance and Leading Process Elongation (A–D) E17 cerebral cortices that were electroporated with the indicated plasmids plus pCAG-EGFP at E14. (A) High-magnification images of the cortical plate (CP), upper IZ (u-IZ), and lower IZ (lo-IZ) are shown. (B) The ratio of cells with the indicated morphology in the IZ, compared with control. Control: *n* = 5 brains, Ncad-sh1023: *n* = 6 brains, L1-shRNA#4: *n* = 8 brains. Significance compared with control was determined by Student's *t* test. **p < 0.01; n.s., no significant differences. (C) The ratio of cells with round morphology in the IZ was significantly increased in the Ncad-sh1023 or L1-shRNA#4-electroporated neurons. Each score represents the mean of ratios ± SEM. Control: *n* = 5 brains, Ncad-sh1023: *n* = 6 brains, L1-shRNA#4: *n* = 8 brains. Significance compared with control was determined by Student's *t* test (Ncad-sh1023: p = 0.000001416, L1-shRNA#4: p = 0.009111). **p < 0.01. (D) Average leading process length of the locomoting neurons in the IZ. Control: *n* = 33 cells, Ncad-sh1023: *n* = 43 cells, L1-shRNA#4: *n* = 57 cells. Each score represents the mean length ± SEM. Significance was determined by Welch's *t* test (Ncad-sh1023: p = 0.000002662, L1-shRNA#4: p = 0.006570). **p < 0.01. (E) Schematics of morphologies of immature neurons electroporated with control or Ncad-sh1023 or L1-shRNA#4- or Cav1-sh490-expressing vectors in the u-IZ (upper cells) and lo-IZ (lower cells). Scale bar: 10 μm in (A).

To examine the roles of N-cadherin and L1 in neuronal maturation, we performed *in vivo* knockdown experiments. Ncad-sh1023 and L1-shRNA#4, shRNA-vectors for N-cadherin and L1, respectively (Kawauchi et al., 2010, Namba et al., 2014), were electroporated into E14 cerebral cortices, and the electroporated brains were fixed at E17. Consistent with our previous report (Kawauchi et al., 2010), a substantial portion of the Ncad-sh1023-electroporated cells exhibited round morphologies at the lo-IZ, where most of the control cells extended immature neurites (Figure 5A). The ratio of cells with round and multipolar morphologies in the IZ were increased and decreased, respectively, suggesting that immature neurite formation and/or maintenance is suppressed in the Ncad-sh1023-electroporated cortices (Figures 5A–5C). Similarly, the ratio of cells with round morphology was slightly but significantly increased in the L1-knockdown cortices (Figures 5A–5C). These data indicate that N-cadherin and L1 are required for the formation and/or maintenance of the immature neurites (Figure 5E). It further suggests that caveolin-1 might be responsible for the downregulation of N-cadherin and L1 to eliminate the immature neurites before the transition to bipolar morphology.

Interestingly, knockdown of either N-cadherin or L1 resulted in the formation of shorter leading processes in the IZ (Figure 5D), indicating that N-cadherin and L1 are required for the leading process elongation in the IZ. Because it is known that the membrane proteins internalized with caveolin-1 are at least in part sorted to the Golgi apparatus or recycling endosomes in non-neuronal cells (Nichols, 2002, Orlandi and Fishman, 1998), the N-cadherin and L1 internalized at the immature neurites may be recycled to the leading processes to promote their elongation (Figure 7).

Most of the N-cadherin- or L1-knockdown cells were unable to migrate into the CP, consistent with previous reports (Jossin and Cooper, 2011, Kawauchi et al., 2010, Namba et al., 2014). However, a few of these cells were observed to enter the CP and extend leading processes, although these processes were sometimes branched (Figure 5A). This appears consistent with a previous study reporting that N-cadherin-knockout neurons exhibit a migration defect but are ultimately able to extend the leading process (Martinez-Garay et al., 2016).

### Caveolin-1-Mediated Regulation of N-Cadherin Is Required for Neuronal Maturation

**Figure 6:**
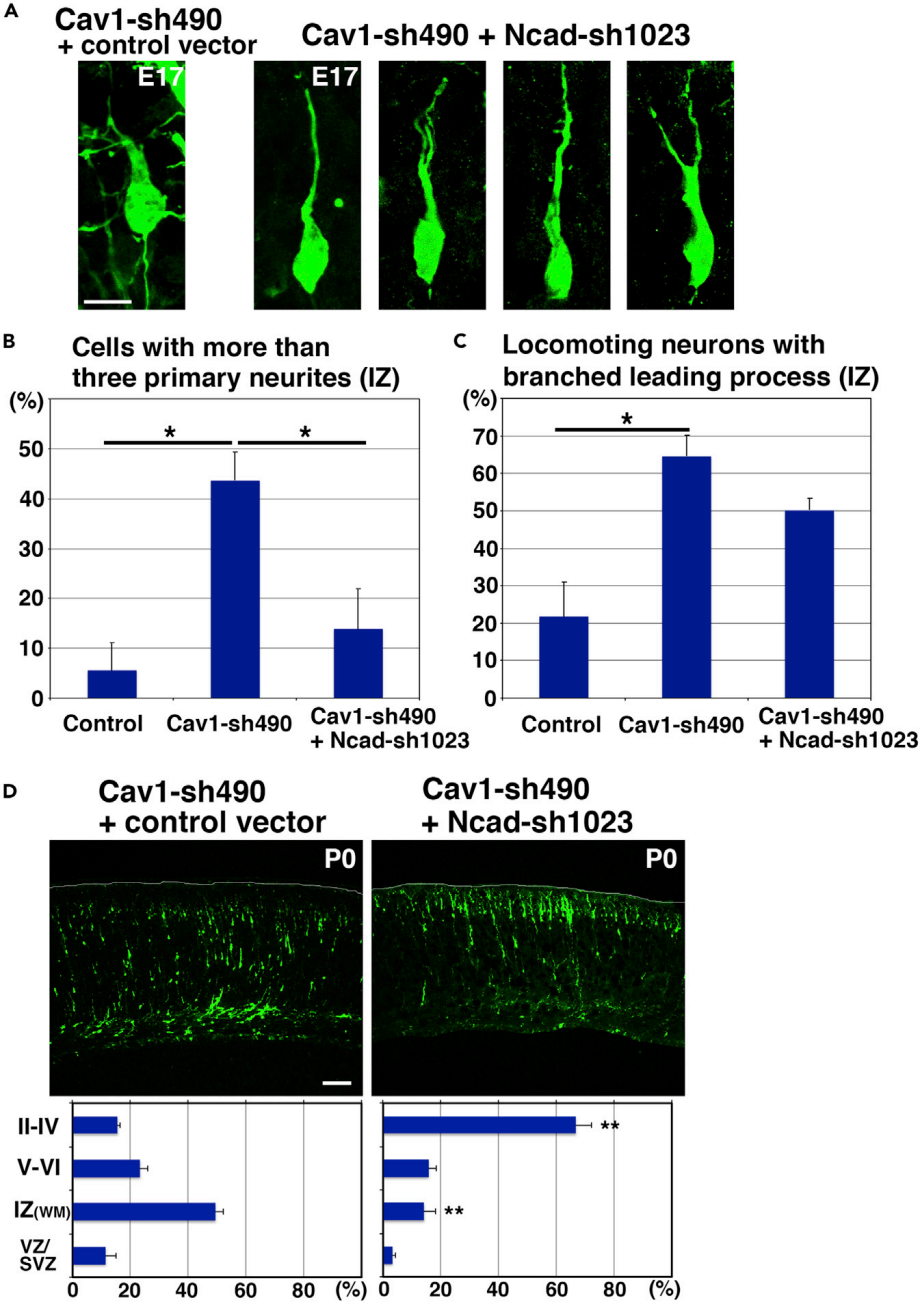
Co-electroporation with Low Concentration of Ncad-sh1023 Restores the Immature Neurite Pruning Defects in the Cav1-sh490-electroporated Neurons (A) Locomoting neurons in the upper IZ of the cerebral cortices at E17, electroporated with the indicated plasmids plus pCAG-EGFP at E14. (B and C) The ratio of locomoting neurons with more than three primary neurites (B) or branched leading processes (C) in the IZ. Control: *n* = 4 (B and C), Cav1-sh490: *n* = 5 (B and C), Cav1-sh490 + Ncad-sh1023: *n* = 5 (B and C). Each score represents the mean ratio or length ± SEM. Significance was determined by Kruskal-Wallis test with post hoc Steel-Dwass test. *p < the critical value at 5% [(B) control versus Cav1-sh490, Cav1-sh490 versus Cav1-sh490 + Ncad-sh1023. (C) control versus Cav1-sh490]. (D) Cerebral cortices at P0, electroporated with the indicated plasmids plus pCAG-EGFP at E14. The lower graphs show the estimation of cell migration, as measured by recording fluorescence intensities of EGFP in distinct regions of the cerebral cortices using Leica SP5 software. Each bar represents the mean percentage of relative intensities ± SEM. Cav1-sh490 + control vector: *n* = 5 brains, Cav1-sh490 + Ncad-sh1023: *n* = 6 brains. Significance compared with Cav1-sh490 was determined by Welch's *t* test [Cav1-sh490 + Ncad-sh1023 (layer II–IV): p = 0.0002388, Cav1-sh490 + Ncad-sh1023 (IZ): p = 0.00007280]. **p < 0.01. See Figures 3B and 3C for the ratio of the number of the electroporated cells in each layer and the multiple comparison data. II–IV, layers II–IV of the cortical plate; V–VI, layers V–VI of the cortical plate; IZ, intermediate zone; WM, white matter; SVZ/VZ, subventricular zone/ventricular zone. Scale bars: 10 μm in (A) and 100 μm in (D).

**Figure 7:**
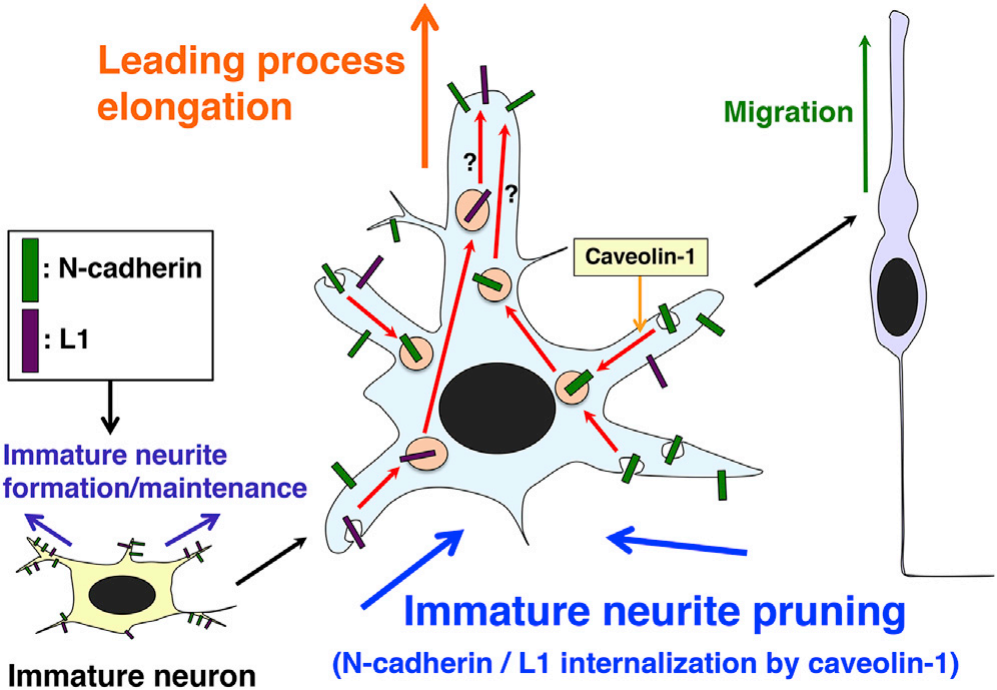
Schematics Depicting Cellular Mechanisms for Immature Neurite Pruning N-cadherin (green rectangles) and L1 (purple rectangles) are required for the immature neurite formation and/or maintenance in the immature cortical neurons (left cell), and therefore should be internalized via caveolin-1-mediated endocytic pathways in the cells with transition from multipolar to bipolar morphologies (middle cell). The internalized N-cadherin and L1 are required for the formation of proper leading process morphologies (middle and right cells).

The aforementioned results indicate that N-cadherin is a major cargo of the caveolin-1-mediated endocytosis in cortical immature neurons. To test whether caveolin-1-mediated internalization of N-cadherin is required for immature neurite pruning, we performed rescue experiments. Cav1-sh490 and a low concentration of Ncad-sh1023 were co-electroporated into E14 cerebral cortices, and the electroporated brains were fixed at E17. Locomoting neurons electroporated with Cav1-sh490 alone extended many abnormal neurites as described above, whereas co-electroporation with Cav1-sh490 and Ncad-sh1023 rescued this phenotype (Figure 6A). The ratio of cells with abnormal primary neurites was significantly restored in the Cav1-sh490- and Ncad-sh1023-electroporated cells (Figure 6B). However, the leading process branching was not completely rescued (Figure 6C), consistent with our data that the formation of proper leading processes requires N-cadherin (Figure 5A). This supports the notion that the N-cadherin that undergoes caveolin-1-mediated endocytosis is recycled to the leading process (Figure 7).

Finally, we examined whether Ncad-sh1023 can rescue the migration defects of the Cav1-sh490-electroporated neurons. At P0, 5 days after electroporation, most of the Cav1-sh490- and Ncad-sh1023-co-electroporated cells reached the superficial layer of the CP, whereas many Cav1-sh490-electroporated cells remained stalled in the IZ (Figure 6D). These data indicate that an excess of surface N-cadherin is one of the main causes of defects in immature neurite pruning and subsequent neuronal migration in Cav1-sh490-electroporated neurons.

## Discussion

Previous studies suggest that caveolin-1 can act independent of caveolae (Head and Insel, 2007), but it was difficult to distinguish between the caveolae-dependent and caveolae-independent functions of caveolin-1. Taking advantage of the fact that caveolae are not found in neurons (Echarri and Del Pozo, 2015, Parton and del Pozo, 2013, Shieh et al., 2011) and that there are no astrocytes containing caveolae (Cameron et al., 1997) in the embryonic cerebral cortices, our present results are able to shed light on the physiological functions of caveolin-1 independent of caveolae.

### *In Vivo* Roles of Caveolin-1 in Brain Development and Its Related Neurological Disorders

Unlike an axon, dendrites arise differently *in vitro* and *in vivo*. Immature neurites become dendrites *in vitro*, whereas *in vivo* the dendrites of cortical neurons originate from a leading process. The immature neurites are eliminated during the early phase of *in vivo* neuronal maturation. Our findings indicate that N-cadherin and L1 are required for immature neurite formation/maintenance and that caveolin-1 promotes the internalization and down-regulation of these cell adhesion molecules to eliminate the immature neurites (Figure 7). Interestingly, N-cadherin and L1 are also required for the formation of proper leading processes, suggesting that internalized N-cadherin and L1 are recycled to the leading processes to promote leading process elongation.

Although several previous reports have shown that caveolin-1 expression levels are low in brain tissues, gene targeting of *caveolin-1* in mice results in altered emotionality, spatial memory, and locomotive activity (Gioiosa et al., 2008, Trushina et al., 2006a), suggesting that a low-level expression of caveolin-1 is required in the brain. In addition, caveolin-1 has been identified as a risk gene for schizophrenia, a neurodevelopmental psychiatric disorder (Allen et al., 2011, Kassan et al., 2017). Because we found that caveolin-1 regulates neuronal maturation *in vivo*, our results support a report suggesting that patients with schizophrenia present with an immature cerebral cortex (Hagihara et al., 2014, Nakao et al., 2017).

### Why Are the *In Vivo*-Specific Maturation Steps Required?

The *in vivo*-specific maturation steps appear important for neurons to find their final destinations, because leading processes that are only observed *in vivo* are thought to have a role in neuronal migration (Bellion et al., 2005, Guerrier et al., 2009, Nishimura et al., 2014, Schaar and McConnell, 2005). Furthermore, the fact that apical dendrites originate from a pia-directed leading process *in vivo*, but not in cultured neurons that do not possess obvious apical dendrites, suggests that *in vivo*-specific maturation steps may also contribute to the pia-directed apical dendrite morphogenesis of cortical pyramidal neurons.

Interestingly, long-distance migration (locomotion mode) appears to be an evolutionary recent migration mode (Nadarajah et al., 2001). Considering that membrane trafficking-related protein families are evolutionarily expanded in humans (Pereira-Leal and Seabra, 2001), membrane trafficking machinery may confer the locomotion mode of migration, which requires immature neurite pruning and leading process formation, to create the mammalian-specific six-layered cortical structures and the pyramidal morphologies of cortical excitatory neurons.

### Possible Roles of Clathrin-Independent Endocytosis

One of the fundamental questions in cell biology is why endocytosis and membrane trafficking pathways are highly diversified. The fact that the same membrane-associated proteins are internalized through several different endocytic pathways further confounds this problem. Clathrin-coated pits were observed at the cytoplasmic dilation (a migrating neuron-specific unique structure found at the proximal region of the leading process) (Bellion et al., 2005, Nishimura et al., 2014, Schaar and McConnell, 2005, Shieh et al., 2011), whereas our results show that caveolin-1 is predominantly expressed in the immature neurites and cell bodies, rather than leading processes, suggesting that each type of endocytosis occurs at distinct membrane regions. In addition, the downstream subcellular trafficking pathway may also be different (Nichols, 2002).

Interestingly, however, a recent report shows that the contribution of clathrin-independent endocytosis to total endocytic flux is limited (less than 5%) in cultured mammalian cell lines (Bitsikas et al., 2014), although many other studies indicate the requirement of clathrin-independent endocytosis (Howes et al., 2010a, Howes et al., 2010b). Our findings indicate that caveolin-1 is strongly expressed in the immature neurons in the IZ, but not in the subsequent locomoting neurons in the CP, whereas a clathrin adaptor subunit, α-adaptin, is broadly expressed throughout the cortex. This suggests that clathrin-independent endocytosis, including caveolin-1-mediated endocytosis, may be applied to spatiotemporally restricted cellular events, such as specific steps of differentiation and maturation, in contrast to clathrin-mediated endocytosis, which may be a major contributor at the basal states.

In conclusion, our findings indicate that caveolin-1 is expressed in immature neurons and regulates clathrin-independent endocytosis of cell adhesion molecules, such as N-cadherin and L1, which is essential for *in vivo*-specific early neuronal maturation in the developing cerebral cortex. Although some caveolin-1 is localized in the GD3 ganglioside-rich membrane domain, future studies are required to determine whether N-cadherin endocytosis occurs within or external to the GD3-rich domain.

### Limitation of Study

Our data indicate that caveolin-1 regulates the internalization of N-cadherin, which is required for the immature neurite pruning in the developing cerebral cortex. Considering that N-cadherin promotes the extension of not only immature neurites but also the leading process and that the majority of the membrane-associated proteins that are internalized via caveolin-1-mediated endocytosis are transported to the recycling endosomes or *trans*-Golgi networks, the internalized N-cadherin may be recycled to the plasma membrane in the tip of the leading process and promote its elongation. However, it is difficult to label the cell surface N-cadherin (or the N-cadherin undergoing caveolin-1-mediated endocytosis) on the immature neurite *in vivo*. Thus, we could not exclude the possibility that newly synthesized N-cadherin, in addition to the internalized N-cadherin, may contribute to the extension of the leading process.

The present work indicates the *in vivo* roles of caveolin-1-mediated endocytosis, but further work is required to clearly delineate spatiotemporal differences between caveolin-1-mediated and clathrin-mediated endocytosis during neuronal maturation in the developing cerebral cortex. Analyzing the detailed localization and upstream regulator(s) of these types of endocytosis may be important to clarify this issue.

## Methods

All methods can be found in the accompanying Transparent Methods supplemental file.

## Supplemental Information

Document S1. Transparent Methods and Figures S1–S8
