# Supplementary material for: Caveolin-1 Promotes Early Neuronal Maturation via Caveolae-Independent Trafficking of N-Cadherin and L1
Source: iScience. 2018 Aug 21;7:53–67. doi: 10.1016/j.isci.2018.08.014 (PMC6135901; doi:10.1016/j.isci.2018.08.014)
Supplement: Document S1. Transparent Methods and Figures S1–S8 [file mmc1.pdf]

ISCI, Volume 7

Supplemental Information

Caveolin-1 Promotes Early Neuronal Maturation via Caveolae-Independent Trafficking of N-Cadherin and L1

Mima Shikanai, Yoshiaki V. Nishimura, Miwa Sakurai, Yo-ichi Nabeshima, Michisuke Yuzaki, and Takeshi Kawauchi

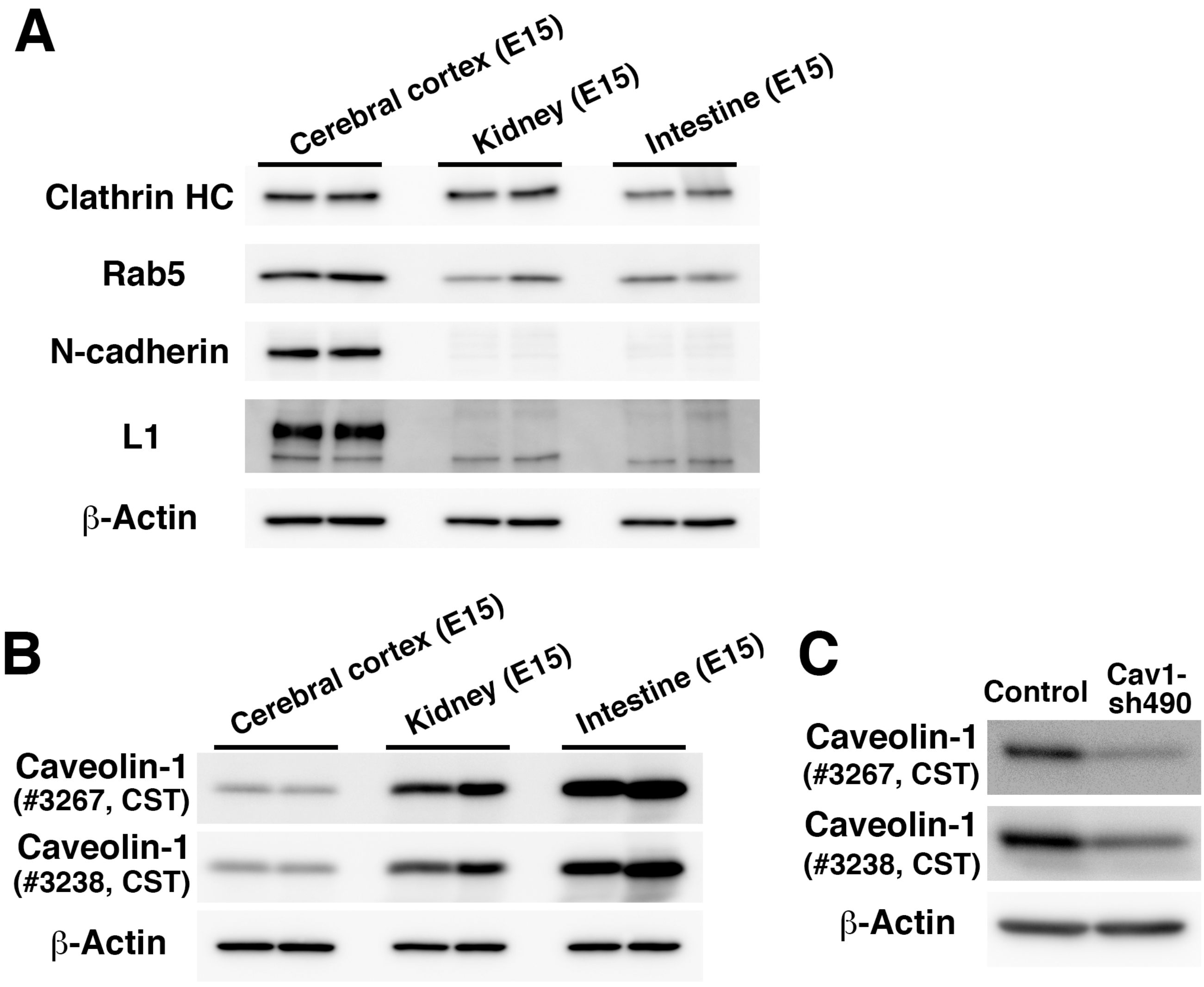

Figure S1. Tissue expression patterns of caveolin-1 and other membrane-associated proteins, Related to Figure 1. (A) Immunoblot analyses of lysates from the indicated tissues at E15 with the indicated antibodies. (B) Immunoblot analyses of lysates from the indicated tissues at E15 with two different antibodies for caveolin-1 (#3267 rabbit monoclonal and #3238 rabbit polyclonal anti-bodies) and anti-β-actin antibody (loading control). The "#3238" is a rabbit antibody that is mainly used in this paper, while the "#3267" is a second anti-caveolin-1 antibody that is used only in this figure for confirmation of the expression of caveolin-1 in embryonic brains. (C) Primary cortical neurons from E15 cerebral cortices were transfected with the indicated plasmids, incubated for two days *in vitro* and subjected to immunoblot analyses of cell lysates with two different antibodies for caveolin-1 (#3267 and #3238) and anti-β-actin antibody (loading control). The data verified that both antibodies clearly detected caveolin-1 because the band intensities are decreased in lysates from caveolin-1 knockdown neurons.

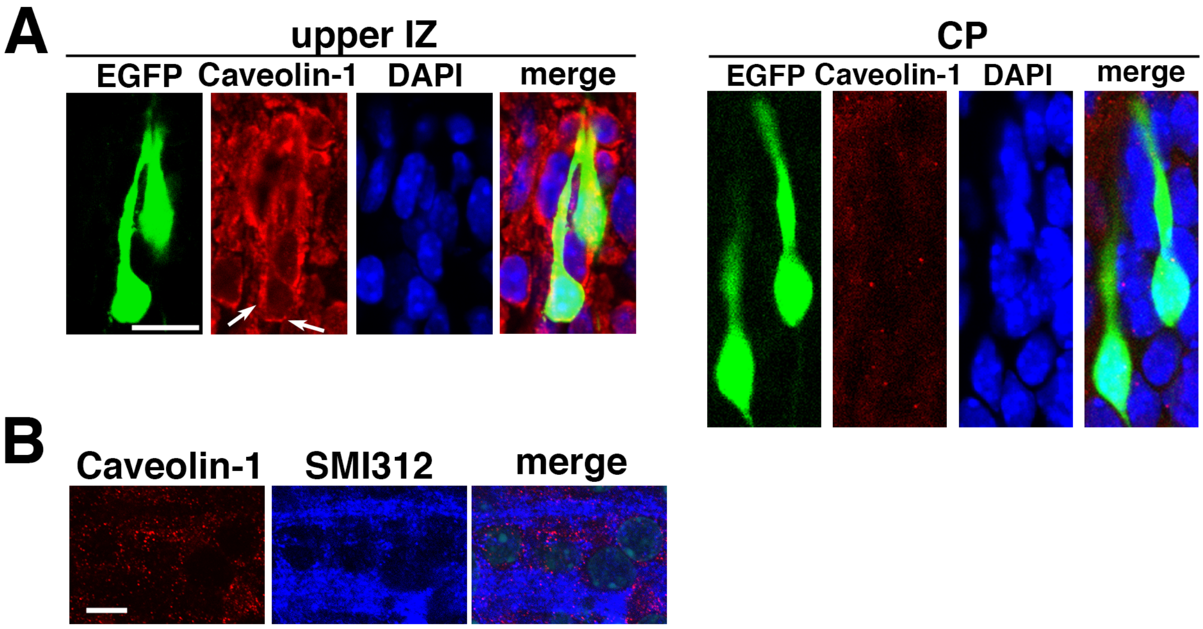

Figure S2. Caveolin-1 expression is low in the locomoting neurons in the CP and the axon bundles in the IZ, Related to Figure 1. (A-B) Cryosections of cerebral cortices at E17, electroporated with pCAG-EGFP at E14, were immunostained with the indicated antibodies and DAPI (blue) for visualizing nuclei. Caveolin-1 expression was observed in the soma of the locomoting neurons in the upper IZ (arrows in A), but not in the CP. SMI312 is an axonal marker. CP: cortical plate, IZ: intermediate zone. The images were obtained with TCS-SP5 (Leica) (A) or A1R (Nikon) (B). Scale bars: 10 μm in (A), 4 μm in (B).

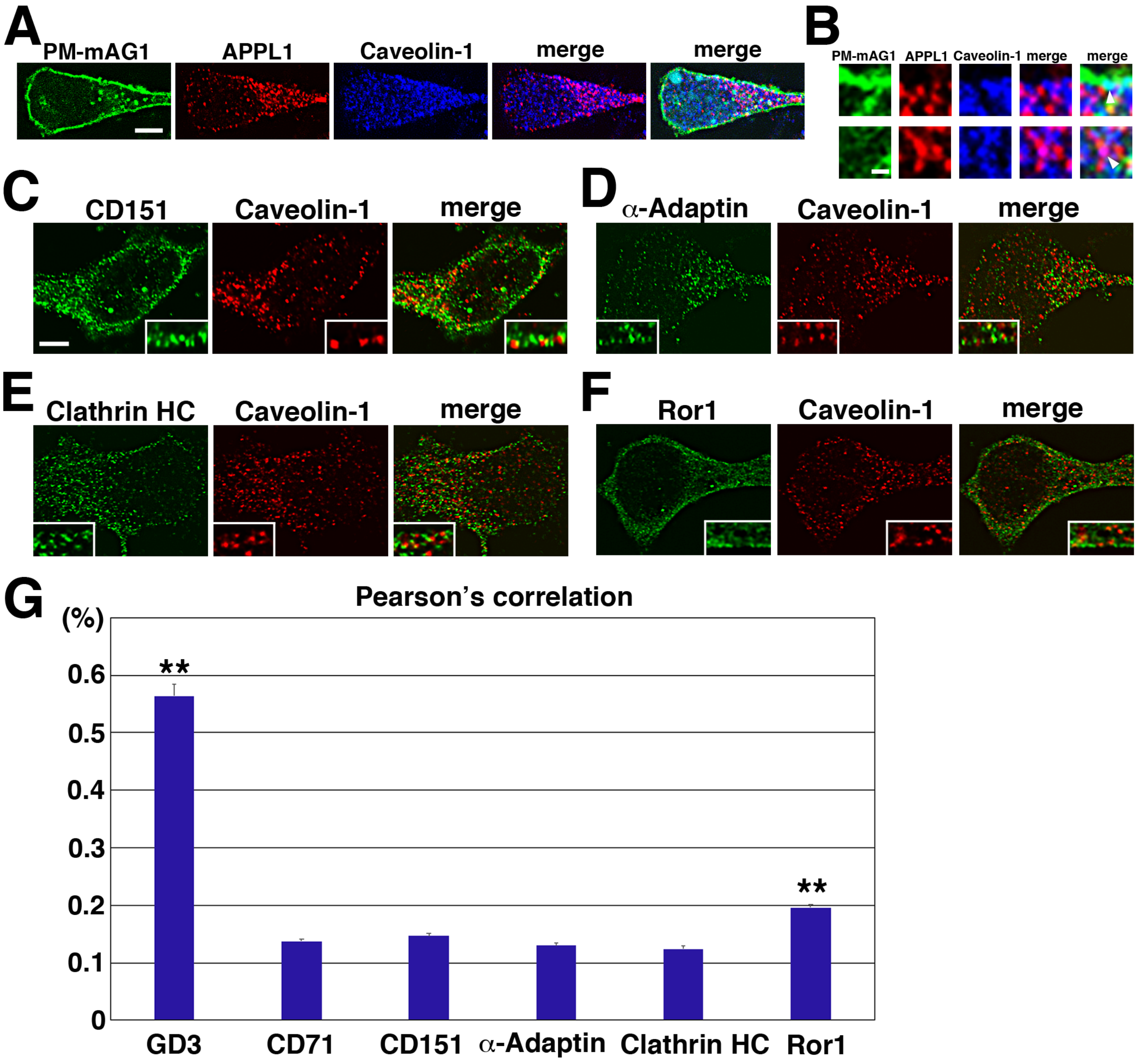

Figure S3. Some localization of caveolin-1 observed in the GD3-rich membrane domains, Related to Figure 1. (A-B) Primary cortical neurons from E15 cerebral cortices were transfected with CAG=PM-mAG1 and incubated for two days *in vitro*. Cells were immunostained with anti-mAG1 (green) and the indicated antibodies. The images were obtained with high-resolution microscopy. Arrowheads in (B) indicate the colocalization of caveolin-1 (blue) and PM-mAG1 (green) (upper panels) or APPL1 (red) (lower panels). (C-F) Primary cortical neurons from E15 cerebral cortices incubated for two days *in vitro* and stained with the indicated antibodies. Insets are high magnification images. (G) The graph shows the colocalization efficient (Pearson's correlation) of the indicated proteins with caveolin-1, as determined using NIS elements software (Nikon). GD3: n = 32 cells, CD71: 48 cells, CD151: 35 cells, α-Adaptin: 61 cells, Clathrin heavy chain (Clathrin HC): 45 cells, Ror1: 51 cells. Each bar represents the mean ratio ± s.e.m. Significance was determined by Steel-Dwass test because the equality of variance was not accepted as determined by Bartlett test. **: < the critical value at 1% (GD3 vs CD71, GD3 vs CD151, GD3 vs α-Adaptin, GD3 vs Clathrin HC, GD3 vs Ror1, Ror1 vs CD71, Ror1 vs CD151, Ror1 vs α-Adaptin, Ror1 vs Clathrin HC). Scale bars: 2 μm in (A), 0.2 μm in (B), 2 μm in (C-F).

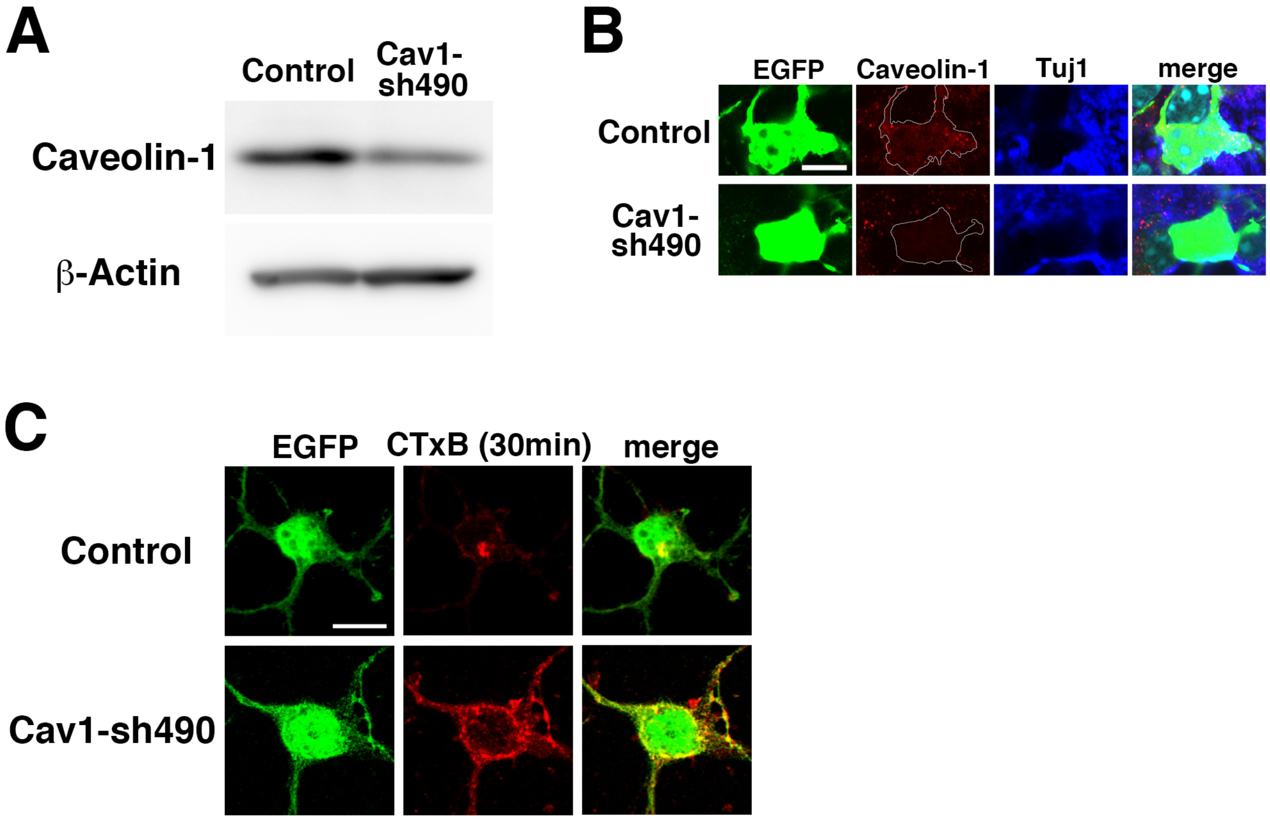

Figure S4. Determination of the knockdown efficiency of Cav1-sh490 and its effect on the internalization of CTxB, Related to Figures 1 and 2. (A) Primary cortical neurons from E15 cerebral cortices were transfected with the indicated plasmids, incubated for two days *in vitro* and subjected to immunoblot analyses of cell lysates with anti-caveolin-1 and anti-β-actin antibodies.
(B) Immature neurons in the IZ of the cerebral cortices at E17, electroporated with the indicated plasmids plus pCAG-EGFP at E14. Frozen sections were immunostained with the indicated antibodies. (C) Primary cortical neurons from E15 cerebral cortices were transfected with the indicated plasmids, incubated for two days *in vitro* and treated with Alexa555-conjugated CTxB for 30 minutes before fixation. CTxB is a potential marker for clathrin-independent endocytosis in cells with high caveolin-1 expression (Singh et al., 2003). Scale bars: 4 µm in (B), 10 µm in (C).

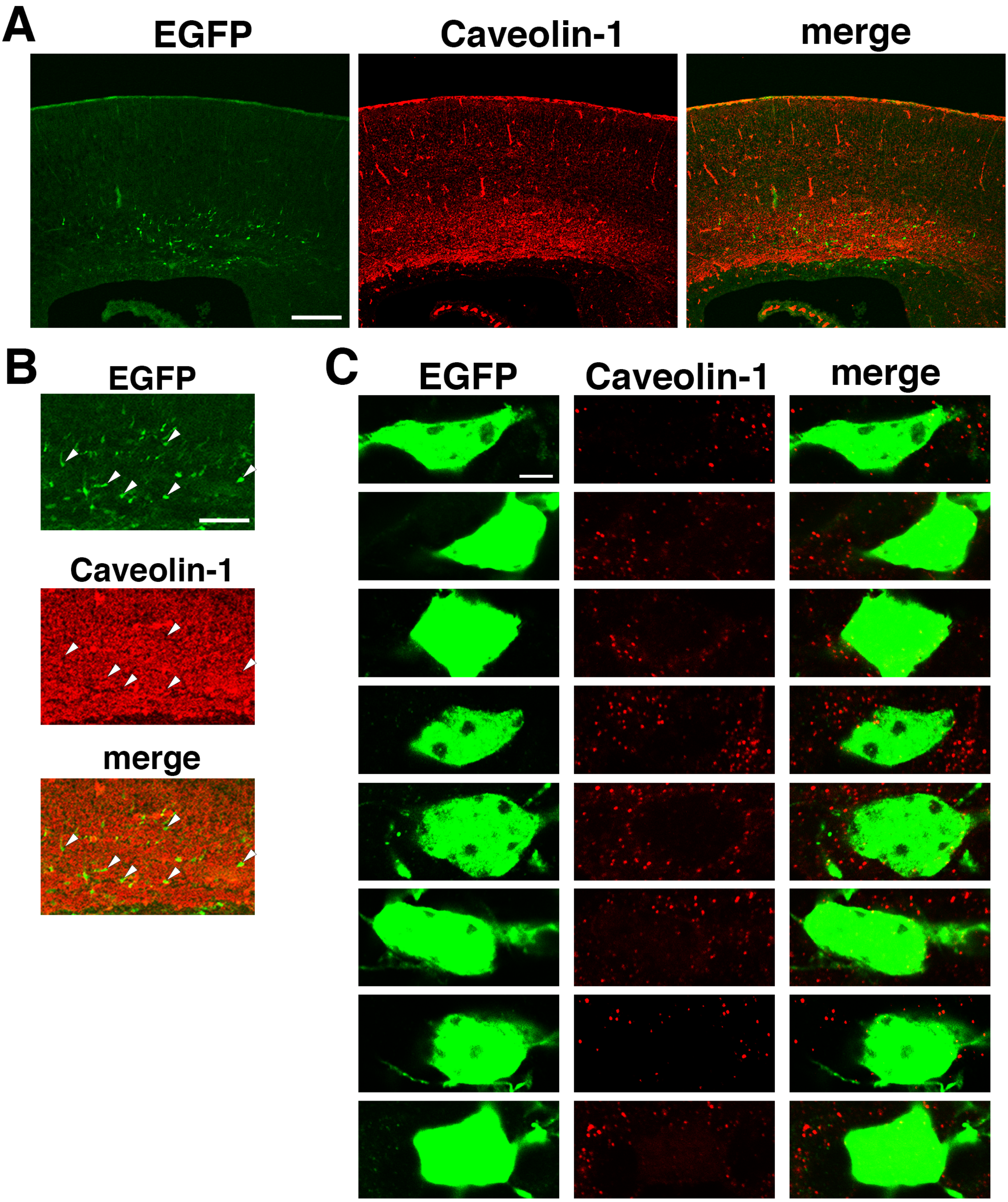

Figure S5. Cav1-sh490 reduces the expression levels of caveolin-1 in immature neurons, Related to Figures 1 and 2. (A-C) Cryosections of cerebral cortices at E17, electroporated with pCAG-EGFP at E14, were immunostained with the indicated antibodies. Staining signals of caveolin-1 (red) are reduced in all Cav1-sh490-electroporated cells (green, arrowheads in B). The images were obtained with FV-10i (Olympus) (A-B) or A1R high-resolution microscopy (Nikon) (C). Scale bars: 200 µm in (A), 100 µm in (B), 2 µm in (C).

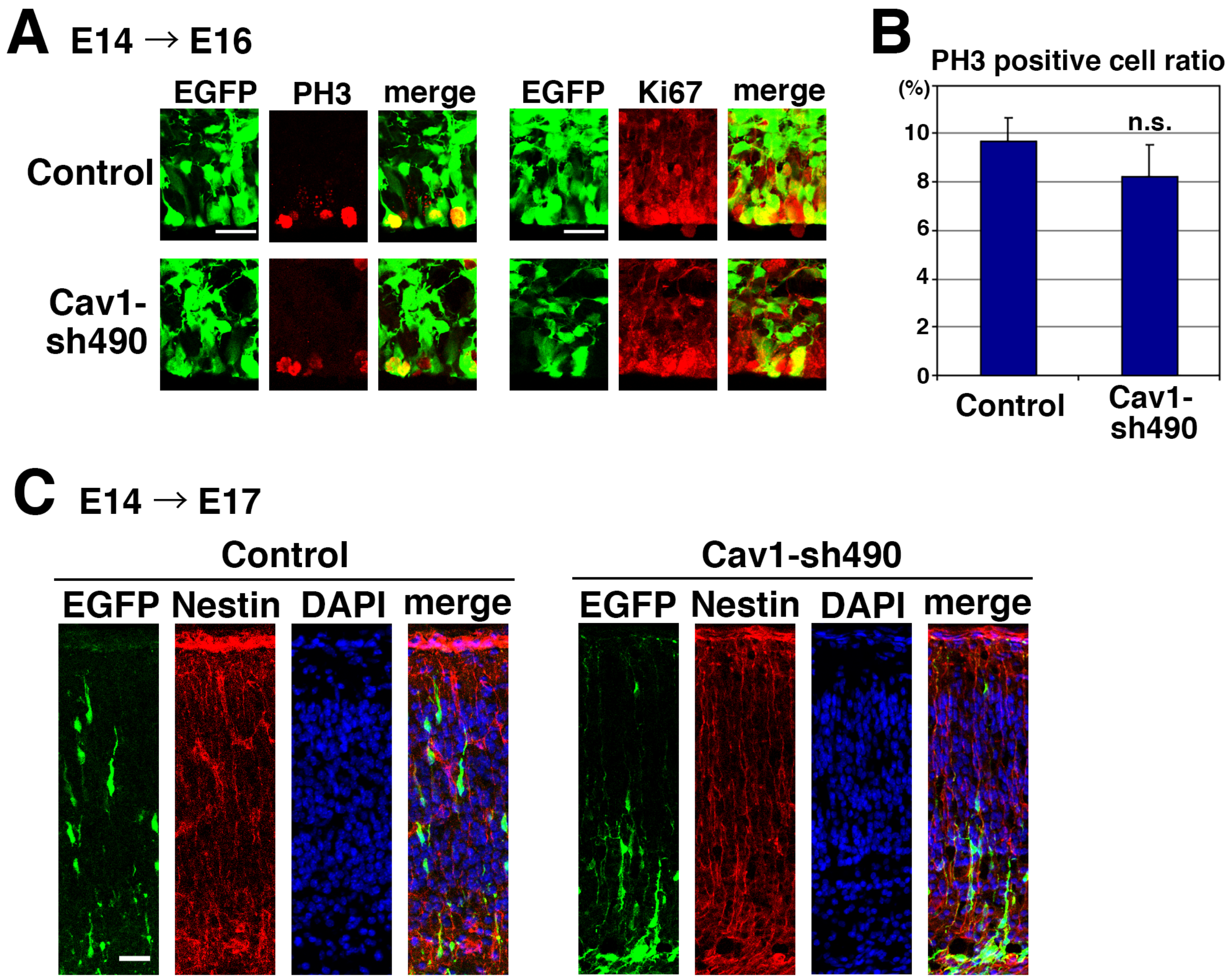

Figure S6. Suppression of caveolin-1 does not affect the proliferation of neural progenitors and radial fiber morphology, Related to Figure 2. (A-C) Cerebral cortices at E16 (46h after electroporation) (A and B) and E17 (C), electroporated with the indicated plasmids plus pCAG-EGFP at E14. Frozen sections were immunostained with anti-EGFP and anti-phospho-Histone H3 (left panels in A) or anti-Ki67 (right panels in A) or anti-Nestin (C) antibodies. The graph in (B) shows the ratio of phospho-Histone H3-positive cells in the electroporated cells in the VZ. Control: n = 4 (477 cells), Cav1-sh490: n = 4 (239 cells). Each bar represents the mean ratio ± s.e.m. Significance compared to control was determined by Student's t test ($P = 0.3982$). n.s.: no significant differences.

Scale bars: 25 μm in (A), 30 μm in (C).

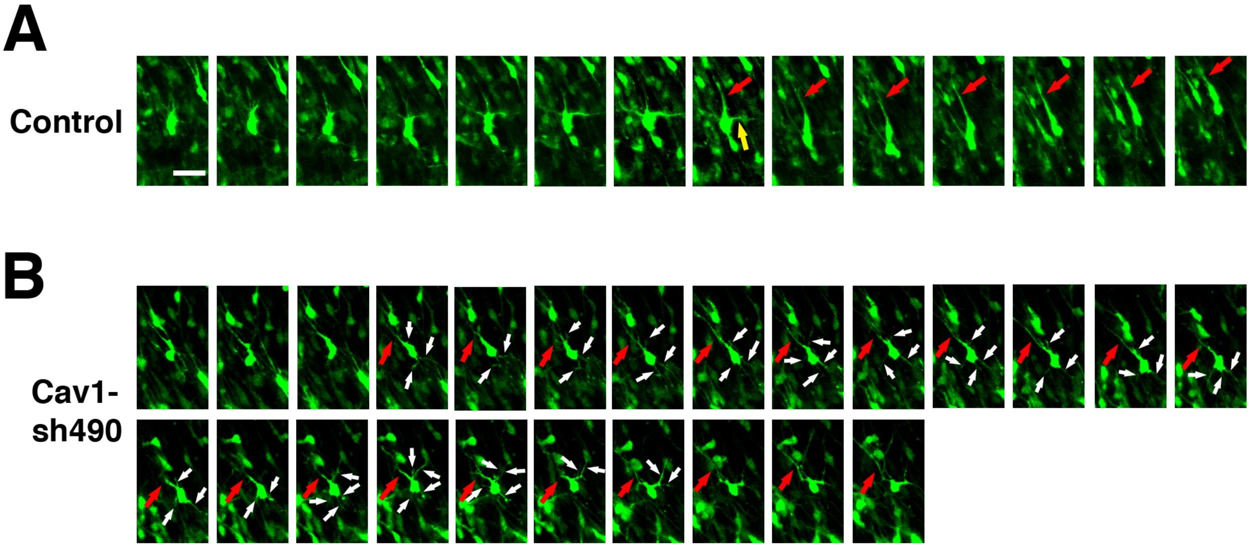

Figure S7. Time-lapse imaging of caveolin-1-knockdown neurons, Related to Figure 2. (A-B) Time-lapse observation of control (A) and Cav1-sh490-electroporated cells (B) in cortical slices from E16 cerebral cortices, electroporated with the indicated plasmids plus pCAG-EGFP at E14. After formation of the leading process (red arrows), control neurons rapidly eliminated their immature neurites (yellow arrow), whereas the caveolin-1-knockdown neurons retained the immature neurites (white arrows) for long periods. Time interval of each frame is 20 min. Scale bar: 20 μm.

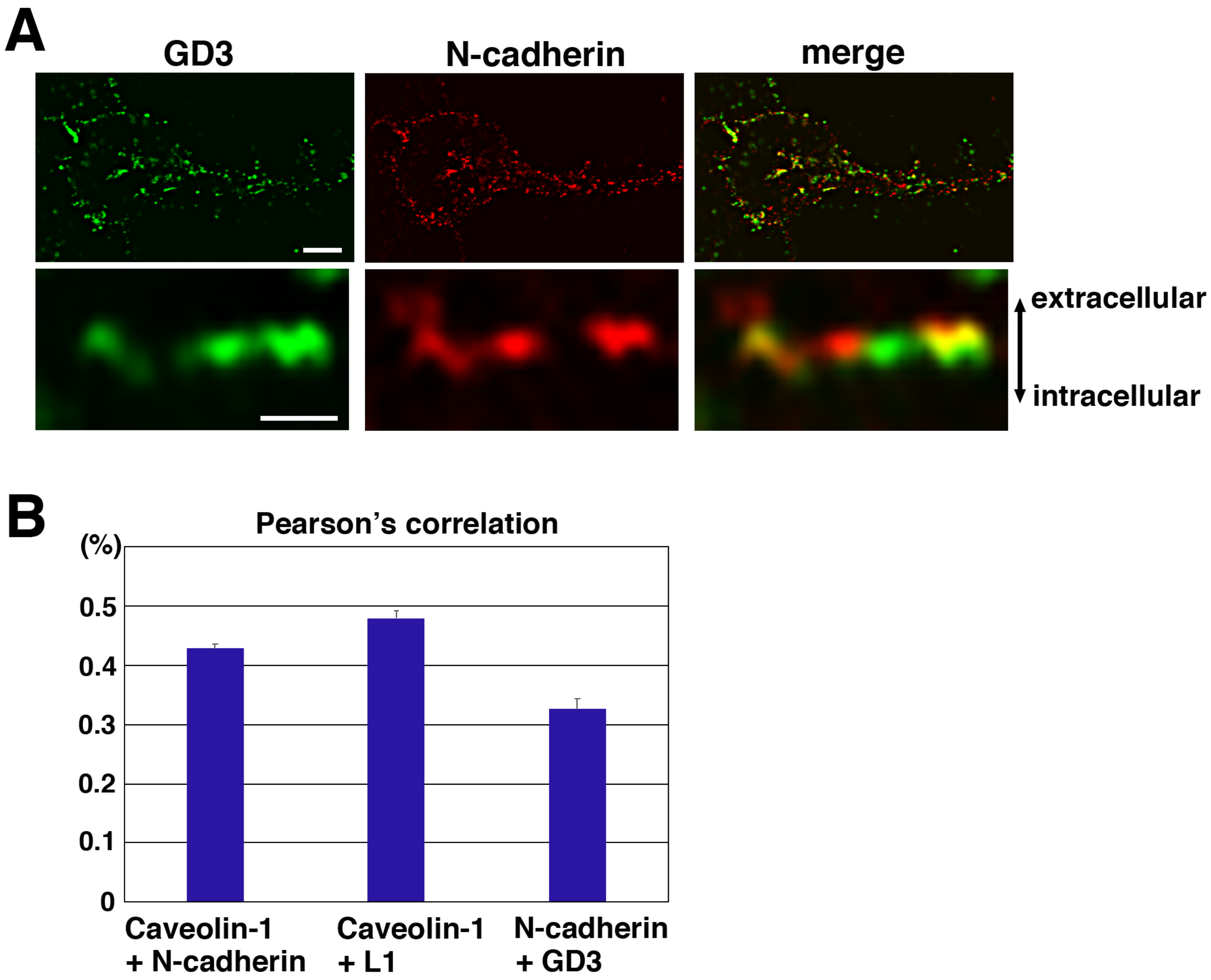

Figure S8. Sparse localization of N-cadherin in the GD3-rich membrane domains, Related to Figure 4. (A-B) Primary cortical neurons from E15 cerebral cortices were incubated for two days *in vitro*. Cells were immunostained with anti-GD3 and anti-N-cadherin antibodies. The lower panels are high magnification images at the cell periphery. The upper and lower sides of the lower panels are the extracellular or intracellular regions, respectively. The images were obtained with high-resolution microscopy. (B) The graph shows the colocalization efficient (Pearson's correlation) of the indicated proteins, as determined using NIS elements software (Nikon). Caveolin-1+N-cadherin: n = 34 cells, Caveolin-1+L1: n = 41 cells, N-cadherin+GD3: n = 31 cells. Each bar represents the mean ratio ± s.e.m. Scale bars: 2 µm in (upper panels in A), 0.25 µm in (lower panels in A).

Transparent Methods

Antibodies and chemical regents

Primary antibodies used in this study were anti-caveolin-1 (3238, Cell Signaling Technology (RRID: AB_2072166); 610406, BD Biosciences), anti-α-adaptin (610502, BD Biosciences), anti-clathrin heavy chain (610499, BD Biosciences), anti-Rab5 (3547, Cell Signaling Technology (RRID: AB_2300649)), anti-APPL1 (3858, Cell Signaling Technology (RRID: AB_2056989)), anti-CD71 (TfR) (13-6800, Zymed), anti-GD3 (ab11779, abcam (RRID: AB_298562)), anti-CD151 (ab33315, abcam (RRID: AB_726140)), anti-Ror1 (H000049, Abnova), anti-N-cadherin (C3865, Sigma; sc-7939, Santa Cruz Biotechnology; ab98952, abcam (RRID: AB_10696943); 13116, Cell Signaling Technology (RRID: AB_2687616)), anti-L1 (ab208155 and ab24345, abcam (RRID: AB_2687508 and AB_448025, respectively)), anti-GFP (A-6455, Molecular Probes; 04404-84, Nacalai; AB16901, Millipore), anti-mAG1 (PM052M, MBL), anti-HA (2367, Cell Signaling Technology (RRID: AB_10691311)), anti-Cavin1 (PTRF) (ab48824, abcam (RRID: AB_882224)), anti-Nestin (556309, BD Biosciences), anti-Phospho Histone H3 (9701, Cell Signaling Technology (RRID: AB_331535)), anti-Ki67 (NCL-Ki67p, Leica), anti-βIII tubulin (Tuj1) (MMS-435P, Covance), SMI312 (837904, BioLegend) and anti-β-actin (A5441, Sigma).
4',6-diamidino-2-phenylindole dihydrochloride solution (DAPI) was purchased from Wako (28718-90-3).

Plasmids

Plasmids were prepared using the EndoFree plasmid purification kit (Qiagen). The attR1-attR2 sequence of the gateway system (Invitrogen) was inserted into pCAG=MCS2 (Kawauchi et al., 2005) and pTα1=MCS1 (Sekine et al., 2011) to generate the destination vectors, pCAG=MCS3GW and pTα1=MCS2GW, respectively. Caveolin-1 cDNA (ORF clone in pENTR221, ID=100003277, purchased from DNAFORM) was inserted into pCAG=MCS3GW and pTα1=MCS2GW to generate CAG=Caveolin-1 and Tα1=Caveolin-1. Plasma Membrane-targeted monomeric Azami-Green 1 (PM-mAG1) (MBL) and ECFP-Mem (Clontech) cDNAs were inserted into pCAG=MCS2 to generate CAG=PM-mAG1 and CAG=ECFP-Mem. L1-shRNA#4 was a generous gift from Prof. Kozo Kaibuchi (Namba et al., 2014). CAG=EGFP, Ncad-sh1023 and CAG=HA-N-cadherin were described previously (Kawauchi et al., 2003; Kawauchi et al., 2010).

To construct shRNA-expressing vectors, oligonucleotides targeting the *caveolin-1* coding sequence (5'-GGCAAGATATTCAGCAACA-3') and its complementary

sequence were inserted into the pSilencer 3.1-H1 vector (Ambion). All contain a hairpin loop sequence (5'-TTCAAGAGA-3'). These sequences were designed based on shRNA sequence analyses (B-Bridge International, Inc). A control vector containing a scrambled non-targeting sequence was purchased from Ambion.

In utero electroporation

Pregnant ICR mice were purchased from SLC Japan or Animal Facility of RIKEN Center for Developmental Biology. Animals were handled in accordance with guidelines established by Keio University, RIKEN Center for Developmental Biology and Institute of Biomedical Research and Innovation. All electroporations in this study were performed on E14 embryos.

In utero electroporation experiments were performed as described previously with minor modifications (Kawauchi et al., 2003). Pregnant mice were deeply anesthetized and an abdominal or right dorsal incision was made to access the uterus. Approximately 1 μl of plasmid DNA (shRNA experiments: 3 μg/μl, low concentration of Ncad-sh1023: 1 μg/μl, rescue experiments: 1-10 μg/μl, pCAG-EGFP: 0.5 μg/μl) in endotoxin-free TE buffer (Qiagen) containing Fast Green was injected into the lateral ventricle of embryonic brains with a glass micropipette (GD-1, Narishige). Holding the embryo *in utero* with forceps-type electrodes (NEPA GENE or BEX), 50 ms electric pulses of 35 V were delivered five times at intervals of 450 ms with a square electroporator (NEPA21, NEPA GENE or CUY21, BEX). After electroporation, the uterus was placed back into the abdominal cavity, allowing embryos to continue developing. At indicated stages, embryos were harvested and coronal sections of electroporated brains were prepared using a cryostat at the level of the rostral half of the hippocampus to observe the dorso-lateral region of the cortices.

Cortical slice cultures

Slice culture of embryonic cerebral cortices was performed as described previously (Nishimura et al., 2010). Embryonic brains at E16, electroporated at E14, were sectioned into 300 μm coronal slices with a microtome (Leica) in DMEM/F-12 1:1 media (Invitrogen). Cortical slices were cultured on the insert membrane (Millipore) in 2 ml of enriched media (100 μg/ml transferrin, 25 μg/ml insulin, 20 nM progesterone, 60 μM putrescine, 10 ng/ml EGF, 10ng/ml bFGF, 5% Fetal bovine serum and 5% Horse serum) (Miyata et al., 2002) in a CO_2/O_2 incubator (37°C, 5% CO_2, 40 or 60% O_2) under confocal laser scanning time-lapse microscopy, FV1000 (Olympus) or TCL-SP2 (Leica).

Immunohistochemistry

Immunohistochemical analyses were performed as described previously with minor modifications (Kawauchi et al., 2010; Nishimura et al., 2014). Embryonic brains were fixed in 4% paraformaldehyde (PFA) in phosphate buffered saline (PBS) for several hours at 4˚C. Frozen cortical sections were washed with PBS, treated with GS-PBS (10% goat serum in PBS) or DS-PBS (10% donkey serum in PBS) containing 0.05% Triton X-100 for 1 h at room temperature (RT) and subsequently incubated with diluted primary antibodies in GS-PBT (GS-PBS containing 0.1% Tween 20) or DS-PBT (DS-PBS containing 0.1% Tween 20) at 4˚C overnight. After three washes in PBS, sections were treated with Alexa488-, Alexa555- or Alexa647-conjugated secondary antibodies (Molecular Probes) diluted in PBS for 1 h at RT, followed by three washes in PBS. The nuclei were stained with DAPI. Fluorescence images were obtained by TCL-SP5 laser scanning confocal microscopy (Leica) or A1R laser scanning confocal microscopy with a high sensitivity GaAsP detector (Nikon) or FV-10i laser scanning confocal microscopy (Olympus). For high resolution images, confocal images were obtained by Nikon A1R using the narrow pinhole size (0.3 or 0.5) and subjected to deconvolution processing with the Richardson-Lucy algorithm in NIS-ER software (Nikon).

For staining with anti-PH3 antibody or anti-Caveolin-1 antibody (only for high resolution images), frozen cortical sections were treated with HistoVT-One (Nacalai) for 20 minutes at 70°C after fixation.

Primary cultures, Transfection and immunocytochemistry

Primary culture of embryonic cortical neurons was performed as described previously with minor modifications (Kawauchi et al., 2006). E15 mouse embryonic cerebral cortices were treated with 0.25% Trypsin-EDTA for 10-15 min at 37 ˚C and dissociated into single cells by gentle trituration. Cells were suspended in 500 μl of Neurobasal medium (Invitrogen) supplemented with B27 (Invitrogen) and 2 mM L-glutamine (Sigma; Invitrogen), and then plated on coverslips or 6 cm-dishes coated with 0.1 or 1 mg/ml poly-D-lysine (Sigma). Cells were incubated at 37 ˚C for two days. Transfections into primary cultures of E15 cerebral cortices were performed using Amaxa mouse neuron nucleofector kit (Lonza) according to the manufacturer’s instructions with some modifications.

For immunocytochemistry, cells were fixed with 4% PFA in PBS for 20 min, permeabilized with GS-PBS or DS-PBS containing 0.15% Triton X-100 for 5 min, and

blocked with GS-PBS or DS-PBS for 30 min at RT or overnight at 4˚C. Primary and secondary antibodies were treated as described above for immunohistochemistry.

BODIPY-LacCer or CTxB uptake assay

E15 cerebral cortices were dissociated and cultured for two days. Primary cultured neurons were incubated with 5 μM BODIPY-FL C5-LacCer (Molecular Probes) in OPTI-MEM medium (GIBCO) for 10 min on ice and further incubated for 30 min at 37˚C. Cells were washed six times with OPTI-MEM containing 1% BSA for 10 min at 10˚C and fixed with 4% PFA. For CTxB uptake assay, primary cultured neurons were incubated with 5 μg/ml Alexa555-conjugated CTxB in OPTI-MEM for 10 min on ice, washed with Neurobasal medium and further incubated with Neurobasal medium for 30 min at 37˚C. After fixation with 4% PFA in PBS for 20 min, cells were subjected to immunocytochemical analyses.

Immunoblotting

Immunoblot analyses were performed as described previously with minor modifications (Kawauchi et al., 2006; Nishimura et al., 2010).

For preparing cell lysates, primary cultured neurons were washed with ice-cold PBS, treated with lysis buffer (20 mM Tris-HCl (pH 7.5), 150 mM NaCl, 1% Triton X-100, EDTA-free Complete protease inhibitor cocktail (Roche, Basel, Switzerland), 10 mM β-glycerophosphate, 50mM sodium fluoride and 1mM sodium orthovanadate) and harvested with a cell scraper. After 1 h incubation on ice, the lysates were sonicated and centrifuged at 5,000 rpm for 5 min at 4°C to remove cell debris. The supernatants were mixed with SDS sample buffer (50 mM Tris-HCl (pH 6.8), 2% SDS, 10% glycerol, 100 mM dithiothreitol (DTT) and bromophenol blue).

Cell lysates in SDS sample buffer were separated with SDS-PAGE and transferred onto polyvinylidene difluoride (PVDF) membranes. Membranes were blocked with 5% skim milk or carbo-free blocking solution (SP-5040, Vector laboratories) in PBST (PBS containing 0.05% Tween20) or TBST (20 mM Tris-HCl (pH7.5), 150 mM NaCl, and 0.05% Tween20) for 1h and probed with primary antibodies in 5% skim milk in PBST or TBST or Can Get Signal reagents (TOYOBO), followed by treatment with horseradish peroxidase-conjugated secondary antibodies and ECL Plus or ECL Prime Western blotting detection reagents (Amersham). Signals were detected and measured with a cooled CCD camera (LAS-3000mini or LAS-4000mini, Fuji-firm) and the Multi Gauge software (Fuji-firm).

Quantitative analysis for the ratio of cells with different morphology

Morphology of the immature neurons in the IZ was analyzed on frozen sections of the cerebral cortices at E17, 3 days after electroporation. The numbers of cells in the IZ with a locomoting or round or multipolar (without a leading process) were counted. Locomoting cells were defined as cells with a thick and pia-directed leading process. Using our definition, both round and multipolar cells do not possess a leading process. Multipolar cells were defined as cells with more than three neurites or polygonal morphology.

Quantitative analysis for the leading process length and branching and the number of primary neurites

The ratio of the locomoting neurons (leading process-containing cells) with a branched leading process or more than three primary processes to the total number of the locomoting neurons in the IZ was determined on frozen sections of the cerebral cortices at E17, 3 days after electroporation. Leading process length of the locomoting neurons in the IZ was measured by the Leica SP5 software.

Quantitative analysis for the neuronal positioning

The extent of migration was estimated by recording fluorescence intensities of EGFP in distinct regions of the cerebral cortices, as described previously (Kawauchi et al., 2003, 2006). Fluorescence images of the frozen sections of the electroporated brains were captured by TCS-SP5 laser scanning confocal microscopy (Leica). Fluorescence intensities within the same width regions in layers II-IV, V-VI, IZ, and SVZ/VZ of the cerebral cortices were measured by Leica SP5 software. Relative intensities to the total fluorescence were calculated and plotted in graphs with standard errors. For cell quantification, cell numbers within the same width regions in layers II-IV, V-VI, IZ, and SVZ/VZ of the cerebral cortices were counted and the ratio of the cell number in each layer to total cell number was calculated and plotted in graphs with standard errors.

Quantitative estimation of cell surface levels of N-cadherin or L1

Primary cortical neurons (2 DIV) were washed with ice-cold PBS and fixed with 4% PFA in PBS for 20 min on ice. After two washes with PBS, cells were blocked with DS-PBS for 30 min at RT and incubated with diluted primary antibody (anti-N-cadherin (C3865, Sigma) or anti-L1 (ab208155, abcam) antibody for surface N-cadherin or L1 staining, respectively) in DS-PBS for 60 min at RT. After three washes in PBS, cells were permeabilized with DS-PBS containing 0.15% Triton X-100 for 5 min at RT, and

incubated with diluted primary antibodies (anti-N-cadherin (sc-7939, Santa Cruz Biotechnology) or anti-L1 (ab24345, abcam) antibody for total N-cadherin or L1 staining, respectively, and anti-GFP chick antibody (AB16901, Millipore)) in DS-PBT at 4°C overnight. Secondary antibodies were applied as described above. After three washes in PBS, cells were treated with Alexa488- or Alexa555-conjugated secondary antibodies (Molecular Probes) diluted in PBS for 60 min at RT, followed by three washes in PBS.

Quantitative estimation of N-cadherin localization on the plasma membrane and early endosomes

Primary cortical neurons were transfected as indicated with CAG=PM-mAG1 and Cav1-sh490 or control vectors. After two days culture *in vitro*, cells were fixed with 4% PFA in PBS for 20 min and subjected to immunocytochemical analyses for anti-mAG1, anti-APPL1 and anti-N-cadherin antibodies. Fluorescence intensities of N-cadherin in the PM-mAG1-positive region (plasma membrane) and APPL1-positive region (early endosomes) in each neuron were measured using NIS elements software (Nikon). The ratio of the fluorescence intensities in the PM-mAG1- or APPL1-positive regions to that of whole cells was calculated.

For *in vivo* analyses, frozen sections of E17 cerebral cortices, electroporated with CAG=PM-mAG1, CAG=HA-N-cadherin and Cav1-sh490 or control vectors at E14, were examined immunohistochemically with anti-HA antibody. Fluorescence intensities of HA-tagged N-cadherin in the PM-mAG1-positive region (plasma membrane) in each neuron were measured using NIS elements software (Nikon). The ratio of the fluorescence intensities in the PM-mAG1-positive regions to that of whole cells was calculated.

Statistical analyses

Data are presented as mean ± s.e.m. Statistical significance was calculated using two-tailed Student’s *t* test (for data showing normal distribution and equality of variance), Welch’s *t* test (for data showing normal distribution, but not equality of variance), Mann-Whitney's U test (for data that are not normal distribution or for categorical data), paired *t* test (for paired sample) or multiple comparison analyses (one way ANOVA with post hoc Tukey-Kramer test (parametric), Dunnett test (parametric) and Kruskal-Wallis test with post hoc Steel-Dwass test (non-parametric)), by using Statcel3 software (OMS). A P value of < 0.05 was considered statistically significant.

Supplemental References

Kawauchi, T., Chihama, K., Nabeshima, Y., and Hoshino, M. (2003). The in vivo roles of STEF/Tiam1, Rac1 and JNK in cortical neuronal migration. EMBO J *22*, 4190-4201.

Kawauchi, T., Chihama, K., Nabeshima, Y., and Hoshino, M. (2006). Cdk5 phosphorylates and stabilizes p27kip1 contributing to actin organization and cortical neuronal migration. Nat Cell Biol *8*, 17-26.

Kawauchi, T., Chihama, K., Nishimura, Y.V., Nabeshima, Y., and Hoshino, M. (2005). MAP1B phosphorylation is differentially regulated by Cdk5/p35, Cdk5/p25, and JNK. Biochem Biophys Res Commun *331*, 50-55.

Kawauchi, T., Sekine, K., Shikanai, M., Chihama, K., Tomita, K., Kubo, K., Nakajima, K., Nabeshima, Y., and Hoshino, M. (2010). Rab GTPases-dependent endocytic pathways regulate neuronal migration and maturation through N-cadherin trafficking. Neuron *67*, 588-602.

Miyata, T., Kawaguchi, A., Saito, K., Kuramochi, H., and Ogawa, M. (2002). Visualization of cell cycling by an improvement in slice culture methods. J Neurosci Res *69*, 861-868.

Namba, T., Kibe, Y., Funahashi, Y., Nakamuta, S., Takano, T., Ueno, T., Shimada, A., Kozawa, S., Okamoto, M., Shimoda, Y., *et al*. (2014). Pioneering axons regulate neuronal polarization in the developing cerebral cortex. Neuron *81*, 814-829.

Nishimura, Y.V., Sekine, K., Chihama, K., Nakajima, K., Hoshino, M., Nabeshima, Y., and Kawauchi, T. (2010). Dissecting the factors involved in the locomotion mode of neuronal migration in the developing cerebral cortex. J Biol Chem *285*, 5878-5887.

Nishimura, Y.V., Shikanai, M., Hoshino, M., Ohshima, T., Nabeshima, Y., Mizutani, K., Nagata, K., Nakajima, K., and Kawauchi, T. (2014). Cdk5 and its substrates, Dcx and p27kip1, regulate cytoplasmic dilation formation and nuclear elongation in migrating neurons. Development *141*, 3540-3550.

Sekine, K., Honda, T., Kawauchi, T., Kubo, K., and Nakajima, K. (2011). The outermost region of the developing cortical plate is crucial for both the switch of the radial migration mode and the Dab1-dependent "inside-out" lamination in the neocortex. J Neurosci *31*, 9426-9439.
